# Comparative Analysis of Regions with Distorted Segregation in Three Diploid Populations of Potato

**DOI:** 10.1534/g3.116.030031

**Published:** 2016-06-23

**Authors:** Norma C. Manrique-Carpintero, Joseph J. Coombs, Richard E. Veilleux, C. Robin Buell, David S. Douches

**Affiliations:** * Department of Plant, Soil and Microbial Sciences, Michigan State University, East Lansing, Michigan, 48824; † Department of Horticulture, Virginia Polytechnic Institute and State University, Blacksburg, Virginia, 24061; ‡ Department of Plant Biology, Michigan State University, East Lansing, Michigan, 48824

**Keywords:** recombination rates, high-dense linkage map, Infinium 8303 potato array

## Abstract

Genes associated with gametic and zygotic selection could underlie segregation distortion, observed as alterations of expected Mendelian genotypic frequencies in mapping populations. We studied highly dense genetic maps based on single nucleotide polymorphisms to elucidate the genetic nature of distorted segregation in potato. Three intra- and interspecific diploid segregating populations were used. DRH and D84 are crosses between the sequenced doubled monoploid DM 1-3 516 R44 *Solanum tuberosum* Group Phureja and either RH89-039-16 *S. tuberosum* or 84SD22, a *S. tuberosum* × *S. chacoense* hybrid. MSX902 is an interspecific cross between 84SD22 and *Ber*83 *S. berthaultii* × 2 × species mosaic. At the 0.05 significance level, 21%, 57%, and 51% of the total markers mapped in DRH, D84, and MSX902 exhibited distorted segregation, respectively. Segregation distortion regions for DRH were located on chromosomes 9 and 12; for D84 on chromosomes 2, 3, 4, 6, 7, and 8; and on chromosomes 1, 2, 7, 9, and 12 for MSX902. In general, each population had unique segregation distortion regions and directions of distortion. Interspecific crosses showed greater levels of distorted segregation and lower recombination rates as determined from the male parents. The different genomic regions where the segregation distortion regions occurred in the three populations likely reflect unique genetic combinations producing distorted segregation.

Distorted segregation, the deviation of the observed genotypic ratios from the expected frequencies based on Mendel’s laws of inheritance, is considered an evolutionary force primarily associated with genetic factors involved in reproduction and fitness (Sandler and Novitski 1957; Taylor and Ingvarsson 2003). Several mechanisms, as well as genes associated with distorted segregation, have been reported in plants. In the meiotic drive system, deleterious alleles or meiotic associated loci cause gametophytic abortion, sterility, or preferential transmission of chromosomal segments or alleles to the germinal cells (Fishman and Saunders 2008; Kanizay *et al.* 2013). Zygotic selection, chromosomal rearrangement, and genomic interactions are also associated with distorted segregation causing zygotic abortion, hybrid sterility, haploid induction, and restriction of gene introgression (Gadish and Zamir 1987; Jiang *et al.* 2000; Moyle and Graham 2006; Rieseberg *et al.* 1995; Xu *et al.* 2013). The type of cross and mapping population can influence the incidence of genomic regions exhibiting distorted segregation (Liu *et al.* 2010). Greater levels of distortion have been found in interspecific compared to intraspecific crosses of various Solanaceae species, as well as in rice doubled haploid (DH) compared to F_2_ populations derived from the same intraspecific cross (Yamagishi *et al.* 2010; Zamir and Tadmor 1986). Genomic interactions causing distorted segregation decreased in near-isogenic lines (NILs) compared with early backcross generations of a tomato interspecific segregating population (Moyle and Graham 2006). In general, the study of segregation distortion has been useful to screen and identify gametophytic mutants affecting male and female fertility in plants (Baumbach *et al.* 2012; Grini *et al.* 1999; Lalanne *et al.* 2004), as well as several hybrid sterility, hybrid weakness, and gametophytic competition genes acting as inter- or intraspecific reproductive barriers (Harushima *et al.* 2002; Lu *et al.* 2002). Gametic and zygotic hybridization barriers, their impact on the process of speciation, and gene flow have critical interest for both plant evolutionists and breeders.

Distorted segregation has been commonly reported in linkage analysis of diploid populations of potato (Bonierbale *et al.* 1988; Felcher *et al.* 2012; Gebhardt *et al.* 1991; Jacobs *et al.* 1995; Kreike and Stiekema 1997; Rivard *et al.* 1996), with percentages of distortion varying from 6–40%. In general, lower proportions of distorted segregation have been observed in intraspecific crosses compared with populations derived from introgression of wild potato species. Differences in chromosomal structure, presence of the self-incompatibility locus, meiotic mutants [genetic variants affecting microsporogenesis or megasporogenesis (Peloquin *et al.* 1999)], and lethal alleles have been proposed as factors influencing distorted segregation in potato (Chani *et al.* 2002; Gebhardt *et al.* 1991; Jacobs *et al.* 1995).

Highly saturated genetic maps facilitate the study of genetic phenomena such as distorted segregation (Harushima *et al.* 1996). The genome coverage and density of markers of these maps allow the identification of segregation distortion regions (SDRs). The SDRs are defined by clusters of markers closely linked to genes causing distortion, as markers with distorted segregation will cosegregate and result in the highest-skewed genotypic frequencies associated with the distorting factor (Lu *et al.* 2002; Tai *et al.* 2000; Zamir and Tadmor 1986). Analysis of SDRs in multiple mapping populations has been used to determine whether zygotic or gametophytic factors are associated and, in some cases, to identify the genes underlying the distorted segregation (Kumar *et al.* 2007; Lu *et al.* 2002; Wu *et al.* 2010; Xu *et al.* 1997). Likewise, SDRs exhibiting similar patterns of distortion at the same chromosomal regions in several species-related populations could lead to the identification of common genetic factors causing distorted segregation (Lu *et al.* 2002). Studies of SDRs are important to understand evolutionary mechanisms triggering reproductive barriers between related species (Li *et al.* 2012). In plant breeding, detection of cross-specific SDR could help overcome limitations for introgression of genes with improved breeding value. As disease resistance genes have been reported to cosegregate with segregation distorters (Li *et al.* 2009; Tonguç *et al.* 2003), understanding the direction and the rate of segregation distortion facilitates estimation of the appropriate population size and strategy that would enable selection of individuals with the desired trait (Li *et al.* 2010; Zhang *et al.* 2010). For example, if the trait of interest is linked to the self-incompatibility locus, the most common distorted segregation factor in potato, then a larger sized population would be necessary to identify progeny segregating the self-incompatibility locus from the trait of interest. The parental allelic identity of the self-incompatibility locus mediates the pollen-pistil incompatibility reproductive barrier that interferes with successful fertilization in selfed and intraspecific crosses (Camadro *et al.* 2004). The self-incompatibility locus (S) codes for an S-ribonuclease and is tightly linked to a S-F-box protein which are expressed in the pistil and the pollen, respectively. A nonself recognition system allows pollen fertilization (Kubo *et al.* 2010).

The objective of this study was to document the distorted segregation phenomenon for an outcrossing, highly heterozygous species (potato) by comparing regions with distorted segregation in three segregating diploid mapping populations. The Infinium 8303 Potato Array was used to genotype and construct highly dense genetic maps for the three mapping populations (Felcher *et al.* 2012). The SNP (single nucleotide polymorphism) unified mapping platform allowed comparison of commonly mapped regions, and showed that differential recombination rates affected the number of saturated zones per map. High collinearly was identified in the five genetic maps, with a total of 693 commonly mapped SNPs. Lower recombination rates were found for interspecific compared to intraspecific hybrid parents, especially when used as the male parent. Cross specific patterns of distortion were evident. The presence of the self-incompatibility locus, deleterious alleles, and incongruity genes are likely candidates driving the distortion.

## Materials and Methods

### Mapping populations

Four parental lines were used to generate three diploid mapping populations: the sequenced doubled monoploid DM1-3 516 R44 (DM 1-3) *Solanum tuberosum* Group Phureja (Paz and Veilleux 1999); two heterozygous breeding lines: a) 84SD22, a hybrid from *S. tuberosum* × *S. chacoense* (Douches and Quiros 1987) and b) RH89-039-16 *S. tuberosum* (RH) hybrid, kindly provided by Dr. Herman J. van Eck, Wageningen University, (Rouppe van der Voort *et al.* 1997; Van Os *et al.* 2006); and c) a wild potato *S. berthaultii* hybrid (*Ber*83) from the Michigan State University Potato Breeding and Genetics Program (pedigree in Supplemental Material, Figure S1). Three mapping populations were developed from crosses between DM 1-3 × RH (DRH), DM 1-3 × 84SD22 (D84), and 84SD22 × *Ber*83 (MSX902). DRH and D84 populations were previously reported by Felcher *et al.* (2012). The numbers of progeny per cross were 96, 130, and 129, for DRH, D84, and MSX902, respectively.

### SNP genotyping and linkage mapping

The parents and progeny of the three mapping populations were SNP genotyped using the Infinium 8303 Potato Array (Felcher *et al.* 2012). DNA was extracted from leaf tissue using the Qiagen DNeasy Plant Mini Kit (Qiagen, Germantown, MD), then quantified using a Quant-iT^™^ PicoGreen^®^ dsDNA Assay Kit (Invitrogen, San Diego, CA) and adjusted to a concentration of 50 ng/μl. DNA (250 ng) was genotyped using the Infinium 8303 Potato Array and the Infinium HD Assay Ultra on an Illumina iScan Reader (Illumina, Inc., San Diego, CA). Fluorescent signals were converted to SNP genotypic classes using the Illumina GenomeStudio 2011.1 software (Illumina, San Diego, CA) and a three cluster custom file.

Segregating SNPs were selected from the raw genotypic data for each mapping population. SNPs were removed from the initial dataset for the following reasons: low quality signal, any missing data, monomorphism, inconsistences or missing data in the SNP genotype calls within two technical repetitions of each parental line, and localization in more than one position in the potato genome sequence version 4.03 (Sharma *et al.* 2013). An additional visual inspection of clustering patterns in GenomeStudio was performed to identify and exclude SNPs potentially located within paralogous loci in the genome.

The biallelic nature of these SNP markers permitted the identification of two segregation patterns in these mapping populations: single parent (female or male) segregation (1:1) or simultaneous segregation of both parents (1:2:1). According to the type of segregation, we coded the segregating SNPs as <lmxll>, <nnxnp>, and <hkxhk> and imported the genotypic data to JoinMap 4.1 software for mapping (Van Ooijen 2006). Redundant markers and individuals were excluded from linkage analysis. Linkage groups were estimated based on a test for independence with a LOD threshold from 3 to 10. Final maps were calculated using the multipoint maximum likelihood mapping algorithm adjusted for cross-pollinated populations (Van Ooijen 2011). Information on the SNP location within the potato genome was also used to identify the linkage groups, and to adjust the direction of the order of loci along the genetic maps according to potato pseudomolecule assembly. The quality of each map was validated by examining the recombination break point patterns in the progeny as well as the nearest neighbor fit (N.N. Fit) values. These criteria allowed the identification of individuals with unexpectedly more recombination events, or SNPs occurring outside their expected positions.

### Segregation distortion study

Distorted segregation, and deviation of the expected 1:1 (homozygous:heterozygous) and 1:2:1 (homozygous:heterozygous:homozygous) Mendelian genotypic class frequencies, was determined by a chi-square test for each SNP marker. Four levels of significance, 5, 1, 0.1, and 0.001%, which corresponded to *P*-values lower than 0.05, 0.01, 0.001, and 0.00001, were used to study distorted segregation. For the D84 and DRH populations, only heterozygous markers in the male parental line were expected to segregate with a 1:1 ratio, whereas for MSX902, three types of loci were segregating: loci segregating with an expected 1:2:1 ratio, when both male and female parents were heterozygous at a SNP locus, and loci segregating with an expected 1:1 ratio when either the male or female parent was heterozygous and the other parent homozygous. SDRs were defined when more than five closely linked markers, exhibiting significant distortion for a threshold α level of 0.1%, clustered at a minimal distance of 5 cM. Patterns and distribution of SDRs along chromosomes were characterized. Thus, two parent chromosome-haplotypes were established based on marker order and linkage phases calculated for loci segregating in individual parental maps. The chromosome-haplotype segregation frequencies were plotted against physical and genetic position and used to characterize the distribution of SDRs.

### Recombination rate variation

Genetic maps are calculated based on the number of recombination events in a segregating population. Comparison of physical and genetic maps reveals variation in recombination rate on a scale of megabases (Mb). The physical position of SNPs in the Infinium 8303 Potato Array was based on potato pseudomolecule assembly version 4.03 (Sharma *et al.* 2013). The total physical length of each map was calculated based on the genomic coverage of mapped loci. The first Mb position of mapped loci per chromosome was subtracted from the last position. The total physical map length was calculated as the sum of the physical map lengths of all 12 chromosomes. The average genome-wide recombination rate was obtained by dividing the total linkage map size in centimorgans (cM) by its corresponding physical map length in Mb for each population. The average chromosome recombination rate in cM/Mb was calculated by dividing the genetic and physical lengths of each chromosome. Variation for recombination rate along chromosomes was also estimated. The genetic position of each marker was plotted against its physical position to generate Marey maps (Chakravarti 1991). Outlier loci in the curve, due to discrepancies in sequential increasing order between the genetic and the physical position, were excluded from the data set. SNPs that were genetically mapped, but did not have assigned positions on any of the 12 chromosomes of the potato pseudomolecule assembly 4.03, were also eliminated from the analysis. Cubic spline interpolations were calculated to obtain smooth and monotonic curves (Yu *et al.* 2001). The recombination rates were estimated as the derivative of the adjusted curve for each chromosome. Variation of recombination rates was characterized by plotting against Mb position along the chromosomes. The calculations and graphs were made using JMP 10 SAS Institute Inc. (Cary, NC, USA).

### Epistatic interactions

Nucleotide coding of loci at unique bin positions was reconstructed for each homologous chromosome for the entire progeny of each mapping population based on linkage phases estimated by JoinMap4.1. All possible pairs of markers were tested for nonrandom association of alleles at unlinked loci using the linkage disequilibrium analysis in Tassel4.0 (Bradbury *et al.* 2007). A *P*-value threshold of 0.005 was established to identify the significant allelic interactions. This threshold was obtained based on the probability distribution of r^2^ from the linkage disequilibrium analysis for all the crosses as done in McDaniel *et al.* (2007).

### Map comparison

Maps of DRH, D84, and MSX902 mapping populations were aligned to check concordance of SNPs mapped on homologous chromosomes. Graphs were constructed using MapChart software (Voorrips 2002).

### Data availability

The authors state that all data necessary for confirming the conclusions presented in the article are represented fully within the article.

## Results and Discussion

### Linkage map construction

Highly dense genetic maps with different frequencies of loci with distorted segregation were compared to detect the effect of these loci on the quality and resolution of the genetic maps (Table 1). Two criteria were used to exclude loci with distorted segregation: a segregation ratio threshold and a chi-square test using an α threshold level of 0.001%. The initial map included all segregating SNPs. A second map, made only for DRH and MSX902 mapping populations, used a segregation ratio threshold of 1:10 and 1:5:5 based on population size, for paternal or maternal and biparental segregation types, respectively. The D84 mapping population did not have markers outside this threshold criterion. A third map was generated with the set of SNPs remaining after excluding distorted segregation based on a 0.001% α threshold. In general, the markers had a wide and similar distribution along the linkage groups when comparing the maps for each mapping population. Map quality in terms of interval distances was not modified, but resolution decreased, especially when distorted loci at 0.001% were excluded. The number of uniquely mapped loci or bins decreased by 4.3% for DRH, 20% for D84, and 3.1% for MSX902. On chromosome 12, the linkage group was split in two new groups when loci were excluded using the 1:10 threshold, and reduced to a small representation when using the 0.001% α threshold. There were considerably fewer mapped SNPs on chromosome 12 for DRH and MSX902, and chromosomes 2, 3, 4, 6, 7, and 8 for D84, than in the other maps, resulting in an overall smaller size of the D84 genetic map. In the three different distortion-mapping conditions for each mapping population, common SNPs were mapped to the same chromosomes in each mapping population, although some rearrangements of SNP order along the chromosomes was observed. However, this SNP position shifting among maps occurred mainly in chromosomes for the combined map of MSX902, which was not present in the individual parental maps. Similarly, the minimal interval distances in the integrated map of MSX902 were smaller than expected, which was not observed in individual parental maps with only maternal or paternal loci.

**Table 1:** Comparison of genetic maps of diploid segregating mapping populations using different levels of loci with distorted segregation

Population*^a^*	Segregation Distortion Level*^b^*	No. Bin Positions	cM	Interval Distance (cM)		
				Mean	Min	Max
DRH	All distortion	414	813.2	2	1.1	11.7
	Ratio threshold	411	797.8	2	1.1	11.7
	α threshold	396	764.3	2	1.1	11.7
D84	All distortion	460	637.9	1.4	0.8	9.3
	α threshold	368	520.3	1.5	0.8	9.3
MSX902	All distortion	798	781.1	1	0.004	8.4
	Ratio threshold	801	774.7	1	0.004	8.4
	α threshold	773	782.9	1	0.004	8.4
MSX902-P1	All distortion	405	799.8	2	0.8	17.9
	Ratio threshold	405	795.1	2	0.8	17.9
	α threshold	405	796.8	2	0.8	17.9
MSX902-P2	All distortion	305	702.1	2.4	0.8	25.7
	Ratio threshold	304	693	2.4	0.8	25.7
	α threshold	283	667.4	2.5	0.8	27.6

No., number; min, minimum; Max, maximum.

a Three maps for the MSX902 mapping population: combined map and individual maternal (P1) and paternal (P2) information only for <lmxll> and <nnxnp> segregation type.

b The ratio threshold was 1:10 for single parent segregation and 1:5:5 for biparental segregation type. Chi-square α threshold was 0.001%. D84 mapping population did not have markers with this ratio threshold.

Three major factors could affect mapping quality: genotyping errors, missing data, and distorted segregation (Hackett and Broadfoot 2003). In this analysis, SNPs with missing data were excluded, and genotype calls were manually evaluated to eliminate any SNP with genotype errors. Confirmation of recombination break points, N.N. Fit-values, and the presence of markers with suspicious linkage in JoinMap4.1 also helped validate map quality. There were no differences in terms of map order and interval distances due to distorted segregation as found by Hackett and Broadfoot (2003). In their study, genotyping errors were the main factor affecting map quality. As we observed in preliminary maps (data not shown), genotyping error created long interval distances in the chromosome flanking regions, joined different linkage groups, and produced rearrangements of SNP order along chromosomes. The multipoint maximum likelihood method adjusted for cross-pollinated populations in JoinMap4.1 used in this analysis has several advantages: a) the use of Gibbs sampling to estimate multipoint recombination frequency for each parent separately, but simultaneously taking linkage phase into account; b) simulated annealing to determine the order of loci using maximum likelihood; and c) spatial sampling to help to determine the missing genotypes and the genotyping errors (Van Ooijen 2011). We also used the test of independence of segregation to calculate the linkage groups; this test is recommended because it is not affected by systematic segregation distortion (Maliepaard *et al.* 1997). JoinMap4.1 estimates the maps of the two parents separately and simultaneously under the constraint that the order of the loci with biparental segregation is the same in both maps. The integrated map is calculated by averaging lengths over anchored segments and by interpolating or extrapolating for markers segregating in one parent only. This process can produce incorrect ordering in some segments. Thus, the few shifts in the order of loci in the combined map of MSX902, in comparison to individual parental maps, were likely due to the manner that JoinMap4.1 generated the combined map. The recombination probabilities calculated by maximum likelihood in the combined map also creates the small interval distances reported.

Since the map order and size were similar among the different distortion-mapping conditions, the map with all segregating loci detected per mapping population was used for further analysis (Table 2). For the two populations with DM 1-3 as a female parent, the genetic size of the map was 813.2 cM for DRH and 637.9 cM for D84. For the MSX902 cross, three genetic maps were generated: a combined map of 781.1 cM, and two separate maps for each parent of 808.1 cM for 84SD22 and 730.3 cM for *Ber*83. The average density of markers, estimated for total number of loci mapped to unique bin positions, ranged from 1–2 cM per SNP with interval genetic distances between 0.004–14.2 cM. The average number of SNPs mapped per chromosome varied from 162 to 254 in the populations, ranging from a minimum of 88 to a maximum of 362 SNPs per chromosome. The Infinium 8303 Potato Array has a genome-wide coverage of 720.6 Mb of the 725.1 Mb of the net sequence assembled in 12 pseudomolecules of potato genome sequence version 4.03. The physical size of DRH, D84, and MSX902 corresponded to 99.3, 99, and 99.2% of genome-wide coverage of the array.

**Table 2:** General characteristics of five genetic maps using all segregating loci identified for three segregating diploid populations

	DRH	D84	MSX902 (Combined)	P1 MSX902 (84SD22)	P2 MSX902 (*Ber*83)
Total mapped SNPs	1948	2348	3043	2227	1847
Unique bin positions	414	460	798	533	469
Recombination events	756	810	1931	1015	916
Map length (cM)	813.2	637.9	781.1	808.1	730.3
Interlocus distance (cM):					
Mean	2.0	1.4	1.0	1.6	1.6
Range	1.1–11.7	0.8–9.3	0.004–8.4	0.004–10.3	0.07–14.2
Map length (Mb)	715.8	713.2	714.8	713.7	704.4
Physical coverage *vs.* DM 1-3*^a^*	99.3%	99.0%	99.2%	99.0%	97.8%
No. mapped SNPs/Chr:					
Average	162.3	195.7	253.6	185.6	153.9
Range	88–259	125–267	145–362	112–262	98–226

No., number; SNP, single nucleotide polymorphism; Chr, chromosome.

a The Infinium 8303 Potato Array has genome coverage of 720.6 Mb of the assembled pseudomolecules version 4.03. This value was used to estimate the physical coverage of each map.

Besides genome-wide coverage, the Infinium 8303 Potato Array provided many polymorphic markers: 1948, 2348, and 3043 for the DRH, D84, and MSX902 populations, respectively. Therefore, small population size and low recombination rates were the main limitations to generate more saturated maps for DRH, D84, and *Ber*83 with 414, 460, and 469 recombination bins compared to 84SD22 in MSX902 with 533. The ultrahigh-density map of potato created with a set of 10,365 AFLP and SSR and a population of 136 individuals had 569 maternal and 549 paternal bin signatures (Van Os *et al.* 2006). The greatest interval marker distances in DRH, D84, and MSX902 varied from 9.3 to 14.3 cM. These gaps could be associated with alternating recombination hot and cold spots on the genome, but also with nonpolymorphic regions in the genome (Van Os *et al.* 2006).

### Comparative analysis of genetic maps

The number of commonly mapped SNPs in the three mapping populations was calculated, and a map comparison was done to check SNP colocalization between homologous chromosomes in the DRH, D84, and MSX902 maps. A total of 4130 SNPs from the 8303 total SNPs on the Infinium Potato Array were mapped in at least one of the three mapping populations, while 693 SNPs were commonly mapped in all the mapping populations (Table 3). Even though differences in recombination rates caused modifications of genetic distances between loci for each mapping population, there was 98.4% (680) concordance in SNP order along chromosomes for the commonly mapped SNPs in all the mapping populations, and two SNPs mapped to different chromosomes in each mapping population. From the 760 commonly mapped SNPs in DRH and D84, two mapped to different chromosomes, and one on chromosome 12 shifted 19.7 cM in order position between D84 and DRH. In DRH and MSX902, 1001 SNPs were mapped in both mapping populations. Four of those SNPs were located on different chromosomes, and 27 SNPs shifted position within neighboring loci (0.02–3 cM) along sequential marker order between mapping populations, two SNPs on chromosome 9 and 11 shifted 23.9 and 7.9 cM, respectively. Comparing D84 and MSX902, 31 of the 2141 commonly mapped SNPs shifted within close positions (0.2–2.6 cM). The final comparative map unified 1612 SNPs that corresponded to unique positions of DRH, D84, and MSX902 genetic maps, and the common maker positions commonly mapped to all mapping populations and any combination of two mapping populations (Figure S2).

**Table 3:** Number of common single nucleotide polymorphic (SNP) markers mapped in three diploid populations of potato

Chr	Total Mapped	Common for All Maps	DRH and D84	DRH and MSX902	D84 and MSX902
chr01	479	103	108	151	252
chr02	413	91	100	114	246
chr03	358	22	25	39	213
chr04	403	65	70	115	151
chr05	243	37	45	47	118
chr06	353	94	104	120	186
chr07	420	51	54	76	235
chr08	287	51	55	61	172
chr09	372	55	59	96	168
chr10	229	44	49	65	110
chr11	275	41	43	48	147
chr12	296	37	46	65	143
Disconcordant	2	2	2	4	0
Total	4130	693	760	1001	2141

Chr, chromosome.

### Distorted segregation

**Figure 1:**
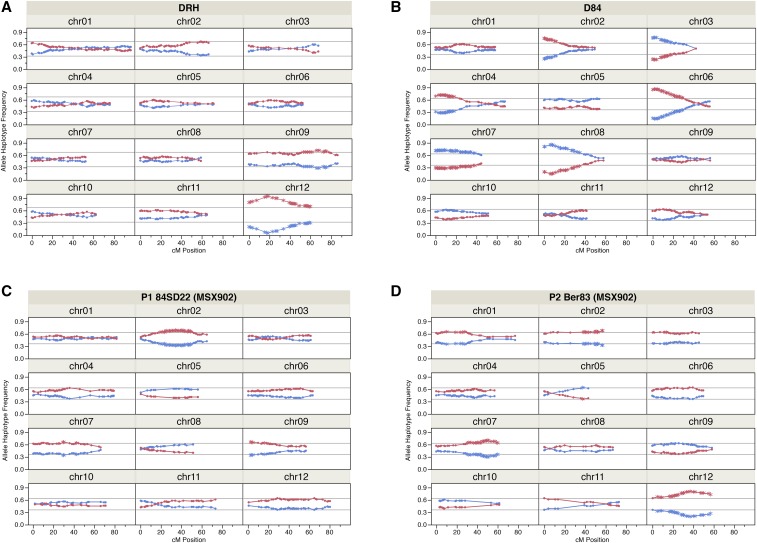
Distribution of segregation ratios of allele haplotypes. (A) RH haplotypes (RH-1 blue, RH-2 red) along the genetic linkage map (cM) of the DRH population. (B) 84SD22 haplotypes (84SD22-1 blue, 84SD22-2 red) along the genetic map of the D84 population. (C) 84SD22 haplotypes female parent P1 (84SD22-1 blue, 84SD22-2 red) along the genetic map of the MSX902 population. (D) *Ber83* haplotypes male parent P2 (*Ber83*-1 blue, *Ber83*-2 red) along the genetic map of MSX902 population. The direction of the order of loci for each chromosomes (chr) is according to potato pseudomolecule assembly 4.03. Single nucleotide polymorphisms (SNPs) are represented by asterisks; those with distorted segregation occur outside of the lines of the confidence interval for a chi-square test with α threshold = 0.1%.

Hybridization may cause interactions involving a wide range of type and levels of genic divergence between the parental forms, due to intrinsic or environmentally mediated incompatibilities (Abbott *et al.* 2013). The proportion of distorted segregation in the progeny of hybrid populations could be considered as an estimation of the level of hybrid intrinsic incompatibilities since, in several cases, this has been reported to be positively correlated with the level of genomic divergence in inter- and intraspecific parents (Hall and Willis 2005). The percentage of loci with distorted segregation in this study was calculated at four levels of significance using the chi-square test (5, 1, 0.1, and 0.001%); the latter considered the proportion of loci with the greatest levels of distortions (Table 4, Table S1, Table S2, Table S3, Table S4, Table S5, and Table S6). At the 5% level of significance, distorted segregation ranged from 21 to 63% for the three mapping populations. There was a wide distribution of loci with distorted segregation at this level of significance, mostly located on chromosomes 1, 2, 9, 11, and 12 for DRH. For D84, loci with distorted segregation occurred on 11 chromosomes with the exception being chromosome 9. The combined map of MSX902 exhibited loci with distorted segregation on all chromosomes. However, based on the segregation type (biparental, maternal, or paternal), loci with distorted segregation were located on: all chromosomes; all chromosomes except chromosomes 1, 3, and 10; and all chromosomes except chromosome 8 for each category, respectively. At the 1% and 0.1% thresholds of significance, the amount of distorted segregation dropped to ranges of 15–45% and 9–39%, respectively. The distribution of distorted segregation at these levels of significance was restricted to fewer chromosomes. For DRH, most of the loci with distorted segregation at the 1% of significance were on chromosomes 2, 9, and 12, whereas at the 0.1% level of significance they were located on chromosomes 9 and 12 (Figure 1A). For D84, chromosomes 2, 3, 4, 5, 6, 7, 8, and 12 exhibited loci with distortion at the 1% of significance, and chromosomes 2, 3, 4, 6, 7, and 8 at the 0.1% level of significance (Figure 1B). For MSX902, loci with distorted segregation were located on all chromosomes at the 1% level of significance, with only a few loci on chromosomes 4, 10, and 11. For the biparental segregation type, they were primarily on chromosomes 1, 2, 5, 7, 8, and 12; maternal segregation type on chromosomes 2, 7, and 12; and paternal segregation type on chromosomes 1, 2, 3, 6, 7, 9, and 12. At the 0.1% level of significance, the distorted segregation spanned chromosomes 1, 2, 5, 6, 7, 9, and 12: on chromosomes 2 and 9 for maternal (Figure 1C); chromosomes 1, 2, 7, and 12 for paternal segregation type (Figure 1D); and chromosomes 2, 5, 6, 7, and 12 for biparental (Figure S3). In general, the lowest proportion of loci with distorted segregation occurred in the DRH, whereas D84 had the greatest proportion of distortion, widely distributed across many chromosomes. The proportion of loci with distorted segregation corresponded with the level of divergence between parents of each mapping population. DRH being an intraspecific cross, MSX902 and D84 interspecific crosses with greater divergence in D84.

**Table 4:** Proportion of loci with distorted segregation at different levels of significance (5, 1, 0.1, and 0.001%) in three diploid populations of potato

Population*^a^*	Segregating SNPs	*P* < 0.05	*P* < 0.01	*P* < 0.001	*P* < 0.00001
DRH	1948	21%	15%	9%	6%
D84	2348	57%	45%	39%	35%
MSX902	3043	51%	35%	15%	4%
<lmxll>	1196	37%	27%	9%	0%
<nnxnp>	816	63%	44%	21%	5%
<hkxhk>	1031	56%	37%	18%	7%

SNP, single nucleotide polymorphism.

a MSX902 population marker segregation type: maternal (<lmxll>), paternal (<nnxnp>), and biparental (<hkxhk>).

The percentage of highly distorted segregation calculated based on the 0.001% threshold of significance ranged between 0 and 35% across the three mapping populations. The loci with highly distorted segregation were located exclusively on chromosome 12 for DRH, chromosomes 2, 3, 4, 6, 7, and 8 for D84, and chromosomes 2, 7, and 12 for MSX902 maps. In addition to the chromosome location, the proportion of mapped loci with distorted segregation also varied among chromosomes. For DRH, all loci on chromosome 12 showed distorted segregation at some level of significance, with 83% in the highly distorted segregation class. For D84, more than 60% of mapped loci on chromosomes 3, 4, 6, 7, and 8 exhibited highly distorted segregation. More than 50% of mapped loci on chromosomes 2 and 12 showed distorted segregation at the 0.1% level of significance for MSX902 for biparental and paternal segregation, while on chromosomes 2 for maternal segregation. On chromosome 12, 40% of mapped loci showed highly distorted segregation.

### Segregation distortion regions

Genetic as well as physical map positions of loci with distorted segregation at a 0.1% significance level were used to define the distribution of SDRs along chromosome maps (Table 5). In heterozygous diploid potato clones, the male or female distorted segregation and allele haplotype with preferential transmission was detected by analyzing those loci where only one parent was segregating. The proportions of homozygous and heterozygous genotypic classes, in conjunction with the linkage phase from the genetic maps, were used to construct the two chromosome haplotypes for each parent and plot their segregation in the progeny along chromosomes (Figure 1 and Figure S3). As expected, the distorted segregation patterns were gradual and smoothly increasing toward the point with the greatest level of distortion. For the DRH and D84 mapping populations, only the male parental line was segregating. Two SDRs were identified for DRH on chromosomes 9 and 12 (Figure 1A). The distortion on chromosome 9 reached a maximum proportion of 1:2.6 that peaked at 68 cM and 56.7 Mb in the genetic and physical map positions, respectively. In a region near the end of the long arm, the SDR spanned a length of 22.4 cM between 53.1–75.5 cM in the genetic map corresponding to 4.3 Mb between 54.1–58.4 Mb in the physical map. On chromosome 12, it was evident that genetic factors throughout the chromosome caused preferential transmission of one chromosome haplotype of RH (designated as RH-2). All SNPs mapped on chromosome 12 showed highly distorted segregation with increasing levels of distortion toward the end of the long arm. The distortion ranged from a ratio of 1:2.2 to 1:18.2, having a maximum peak of distortion at 4 Mb. DRH had the lowest proportion of loci and fewer chromosomes with distorted segregation, but the greatest distorted segregation ratio.

**Table 5:** Range of distorted segregation regions (SDRs) in genetic (cM) and physical (Mb) distances along chromosomes (Chr) identified in three diploid populations of potato

Chr	Distance	DRH	D84	MSX902 (Combined)	P1 MSX902 (84SD22)	P2 MSX902 (*Ber*83)
chr01	cM			19.1–30.9		10.2–27.8
	Mb			61–67.6		61–67.6
chr02	cM		0–11.7	14.2–58.5	18.9–59.2	38.5–57.1
	Mb		6.1–29.4	25.8–47.6	25.8–47.3	36–47.6
chr03	cM		0–18.0			
	Mb		1–51.2			
chr04	cM		0–18			
	Mb		0–62.4			
chr05	cM					
	Mb					
chr06	cM		0–28.9			
	Mb		0.2–51.5			
chr07	cM		0–38.3	22.5–62.6		30.9–60.0
	Mb		0.4–55.3	43.1–56.6		43.1–56.6
chr08	cM		0–34.8			
	Mb		0.3–51.1			
chr09	cM	53.1–75.5		0–2.4	0–4.7	
	Mb	54.1–58.4		0–0.7	0–0.7	
chr10	cM					
	Mb					
chr11	cM					
	Mb					
chr12	cM	0–59.7		9.7–68.1		5.1–56.8
	Mb	0.2–61.1		2–60.5		2–60.5

Chr, chromosome.

The D84 mapping population had six SDRs on chromosomes 2, 3, 4, 6, 7, and 8 (Figure 1B). For chromosomes 2, 3, and 4, the distortion spanned smaller regions in the distal border of the short arm of those chromosomes, reaching levels of distortion of 1:3.0, 1:3.4, and 1:2.6, respectively. The SDRs on chromosomes 6, 7, and 8 cover greater length with similar patterns of distortion toward the distal end of the short arm of each chromosome. Chromosomes 6 and 8 have the greatest proportions of distortion of 1:6.4 and 1:6, respectively. Almost all SNPs on chromosome 7 showed distorted segregation with a maximum proportion of distortion of 1:2.7, thus causing preferential transmission of one of the entire chromosome haplotypes of 84SD22 (designated 84SD22-1).

In the case of the MSX902 mapping population, SDRs were identified not only from each parental line but also due to the influence of segregating biparental loci (Figure 1, C and D and Figure S3). The biparental loci with distorted segregation found on chromosomes 2, 7, and 12 were partially situated within the location of distorted segregation of maternal and/or paternal loci. However, those on chromosomes 5 and 6 did not correspond to regions with distorted segregation of the parental loci. When analyzing the four possible combinations of two homologs, chromosome haplotypes of each parent, there was a tendency for preferential transmission of one combination of chromosomal haplotype or haplotype region influenced by parent segregation. Taking in account only distorted segregation driven by maternal and paternal loci, we identified a total of five SDRs in the combined map on chromosomes 1, 2, 7, 9, and 12. The SDR located in the short arm of chromosome 1 came from the male parent, with a length of 11.8 cM and 17.6 cM for the MSX902 and *Ber*83 genetic maps, and 6.6 Mb for both physical maps. The maximum ratio of distortion was 1:1.9. On chromosome 2, both parental lines showed distortion along the chromosome causing preferential transmission of one haplotype per parent. 84SD22 reached the threshold for distorted segregation in a long region (40.3 cM and 21.5 Mb) with proportions up to 1:2.2, while for *Ber*83, the SDR was confined to a 18.6 cM and 11.6 Mb region on the distal long arm with ratios of 1:2. In the combined map, the region was located between 14.2–58.5 cM and 25.8–47.6 Mb. On chromosome 7, distortion occurred along the entire chromosome for both parental lines, reaching significance only for *Ber*83 in a region of 29.1 cM and 13.5 Mb, and at 40.1 cM and 13.2 Mb in the MSX902 map. This region was located in the distal part of the long arm with a maximum ratio of 1:2.4. On chromosome 9, the SDR was detected in 84SD22 segregation spanning from 0.4–4.7 cM and 0–0.7 Mb, and 2.4 cM length in the MSX902 map. The maximum ratio found was 1:1.9. On chromosome 12, we observed the greatest levels of distortion for this mapping population. For *Ber*83 loci, the distortion occurred along the entire chromosome, reaching levels of distortion of 1:3.9 toward the distal end of the long arm. Using the proportion of segregation along two allele haplotypes per parent, we calculated the segregation of four possible chromosome combinations in the progeny (Figure S3). The distorted segregation in both parental lines produced preferential transmission of one combination on chromosomes 2 and 7, and two combinations on chromosome 12 based on a chi-square test using a threshold of 0.1%.

Few patterns of distorted segregation were common among mapping populations. On chromosome 2, SDRs were detected for D84 and MSX902 in unique regions, whereas the DRH and *Ber*83 MSX902 male maps shared a similar region and direction of distortion even though the distortion was not significant for DRH at 0.1%. The 84SD22 maps from D84 and MSX902 crosses showed similar patterns of distorted segregation only on chromosome 7, and in this instance, the distortion was not significant for D84 at 0.1%. For chromosome 7, the distorted segregation of the *Ber*83 map was in the opposite direction. Chromosome 12, with strong patterns of distorted segregation along the chromosome for DRH and *Ber*83, showed contrasting direction and peaks of distortion.

Different biological factors could be associated with distorted segregation. Genetic factors driving deleterious mutations or causing preferential transmission of alleles or chromosomes through male or female gametes known as gametic or prezygotic selection (Fishman and Saunders 2008; Kanizay *et al.* 2013; Lyttle 1991), or genetic factors mediating selective fertilization and plant developmental fitness classified as zygotic or postzygotic selection (Gadish and Zamir 1987; Jiang *et al.* 2000; Moyle and Graham 2006; Rieseberg *et al.* 1995; Xu *et al.* 2013).

In *Solanum* species within section Petota, different internal hybridization barriers (pollen-pistil incompatibility, nuclear-cytoplasmic male sterility, and the endosperm) have been studied (Camadro *et al.* 2004, 2012). The pollen-pistil incompatibility reproductive barrier interferes with successful fertilization in self-, intra-, and interspecific crosses. The incompatibility sites include the stigma and the first, second, and last third of the style. Incongruity, the lack of genetic information in one parent for some critical characters in the other that produces no functional interaction between two partners (Hogenboom 1979), could cause preferential transmission of one of the alleles in an interspecific cross, but also reduce the production of hybrids from incompatible crosses when hybridization barriers are incomplete. In self-pollinations and backcrosses, the identical allelic configuration of the self-incompatibility locus in the parents has driven distorted segregation on chromosome 1 by producing the absence of specific genotypes in the progeny (Gebhardt *et al.* 1991; Jacobs *et al.* 1995; Rivard *et al.* 1996). Both the presence of loci with sublethal as well as meiotic mutant alleles were also reportedly linked to regions with distorted segregation on chromosomes 10 and 8, respectively (Jacobs *et al.* 1995). These correspond to the recessive *crcr* “crumpled” morphological mutation [stunted plant with contorted stems and crumpled leaves that died a few weeks after germination (Jongedijk *et al.* 1990)] and the *ds1ds1* desynaptic meiotic mutant (affecting the development of normal gametes). The cross, described by Jacobs *et al.* (1995), with a mainly *S. tuberosum* Groups Tuberosum and Phureja genetic background, allowed the occurrence of nonviable recessive homozygotes for sublethal loci identical by descent, thus producing distorted segregation by zygotic selection.

In general, SDRs reported in this study were detected in parent-specific segregation. Due to the homozygosity of the DM 1-3 female parent in DRH and D84 populations, only SDRs from the male parent were analyzed. Two SDRs on chromosomes 9 and 12 were identified in DRH. Examination of the allelic configuration of the RH haplotype segment, with the greatest distorted segregation ratio 1:15–1:18, revealed a block of 11 loci spanning 7 cM corresponding to 0.4 Mb units of RH haplotype 1 that was similar to the DM 1-3 haplotype, except for one locus on position 7. This segment, which would have resulted in homozygous progeny at this region, was significantly underrepresented in the mapping population (0.06%). Similar results were found for a 12-locus segment of 12.8 cM and 1.7 Mb on chromosome 9 for RH haplotype 1 with up to 1:2.6 distorted segregation ratio. The assumption of homozygous recessive alleles with sublethal effects on zygotic viability is unlikely for the strong selection observed on chromosome 12, since DM 1-3 is viable in the homozygous combination. However, meiotic mutations or deleterious alleles producing male gamete abortion or sterility could be associated, taking in account that DM1-3 is male sterile and the progeny could have inherited similar nuclear-cytoplasm interactions at the female parent. Abbott *et al.* (2013), proposed that the genetic divergence underling hybrid attributes that reduce or increase fitness, due to the creation of genetic combinations that have not been tested by selection in parental populations, could be due to similar mechanisms. Unlike the classic Dobzhansky–Muller genic speciation model, independently proposed by several authors (Bateson 1909; Dobzhansky 1937; Muller 1942), where negative effects of a single locus or epistasis among two or more genes allow hybrid inviability or sterility, two more broadly occurring mechanisms could be considered to interpret novel hybrid phenotypes in populations with divergent parental lines: a) additive effects of alleles fixed in different directions, where a novel phenotype depends upon combination; and b) interactions (dominance or epistasis) between alleles fixed independently in divergent parents. The strong deviation against the homozygous genotype on chromosome 12 fits a single-locus selection model or incongruity with negative fitness effects, while gametic competition or fitness advantage genes could be associated with the preferential transmission of heterozygous combinations with RH haplotype 2 to the progeny on chromosome 9.

Several regions and chromosomes affected by distorted segregation have been reported in diploid populations of potato (Bonierbale *et al.* 1988; Felcher *et al.* 2012; Gebhardt *et al.* 1991; Jacobs *et al.* 1995; Kreike and Stiekema 1997). The cross D84 showed the greatest number of SDRs, on chromosomes 2, 3, 4, 6, 7, and 8. The regions and patterns of distortion were consistent with those reported by Bonierbale *et al.* (1988) in a similar cross between *S. phureja* × (*S. tuberosum* × *S. chacoense*). In that cross, the clusters of loci with distorted segregation for the male parent were located on chromosomes 1, 6, 7, 8, and 10. The greatest ratios of distortion were on chromosomes 6 and 8 (1:8 and 1:3.3, respectively), as reported in this study for the D84 mapping population. In both crosses, the male parent was an interspecific hybrid with 50% *S. chacoense* in its pedigree. The D84 mapping population, compared to DRH and MSX902, is the most divergent interspecific cross. In *Solanum* species, a large extent of genome and gene order or synteny is conserved, as was also confirmed by the high collinearity among maps in this study. Therefore, chromosomal rearrangements could not be considered as the potential cause of distorted segregation. Interspecific incongruity in pollen guidance and compatibility interactions could be affecting successful fertilization, thus engendering more SDRs. Broad sets of signals secreted by the female tissue are responsible for pollen guidance; moreover, pollen-associated molecular patterns are generated from pollen-pistil interaction depending on pollen compatibility (Dresselhaus and Franklin-Tong 2013).

In the MSX902 cross, five SDRs were identified on chromosomes 1, 2, 7, 9, and 12. The *Ber*83 parent showed a SDR on chromosome 1 with preferential transmission of a segment of *Ber*83 haplotype 2. In this instance, the self-incompatibility locus could have been associated with the SDR since *Ber*83 has 84SD22 and *S. tuberosum* Group Phureja in its pedigree. The patterns of segregation showed strong and specific constraint on genotype combinations with *Ber*83 haplotype 1 for SDRs on chromosome 1 and 12. On the other hand, the concordance of biparental loci with distorted segregation and SDR for maternal and paternal loci on chromosomes 2, and paternal on chromosomes 7 and 12, suggest additional association of distorted segregation with zygotic selection produced by recessive homozygous loci with sublethal effects. Reciprocal crosses will allow the differentiation of male or female-specific effects of each parent, and the elucidation of whether gametic or/and zygotic selection produce distorted segregation as reported by Fishman and Willis (2005). In a cross between a self-incompatible and self-compatible diploid potato line where the male parent was comprised of half *S. chacoense* (Hosaka and Hanneman 1998), Hosaka and Hanneman likewise observed segregation distortion for seven markers surrounding the *Sli* (*Self-incompatible locus inhibitor*) gene on the distal arm of chromosome 12. They suggested a gametophytic advantage of pollen carrying the *Sli* gene. The male parent of our MSX902 mapping population with a *S. chacoense* genetic background generated similar distortion segregation.

Multiple allelic epistatic interactions between unlinked loci were identified for DRH, D84, and MSX902 crosses [149, 499, and 1680, respectively (Table S7, Table S8 and Table S9)]. The two-loci significant interactions occurred in 14, 16, and 48 different chromosome combinations. Several patterns of interactions were observed. Multiple locus positions along one chromosome mainly interact with a single locus on another chromosome, several random specific two-loci interactions along a two-chromosome combination, or few loci with a significant two-locus interaction between two chromosomes. In most cases, neither of the interactions was located between two regions with distorted segregation, nor toward the point with the greatest distorted segregation, except for D84, where multiple two-locus interactions (88) between chromosomes 4 and 8 were located in the zone with the greatest distorted segregation in both chromosomes, 0–18 and 6–9 cM, respectively. These were also part of the most significant interactions (*P*-value < 0.0001). For MSX902, two-loci interactions were detected within chromosomes from the same parent as well as between parents. These results confirm a wide set of genomic interactions, some on SDR potentially causing preferential transmission of some allelic combinations.

### Recombination rates along chromosomes

**Figure 2:**
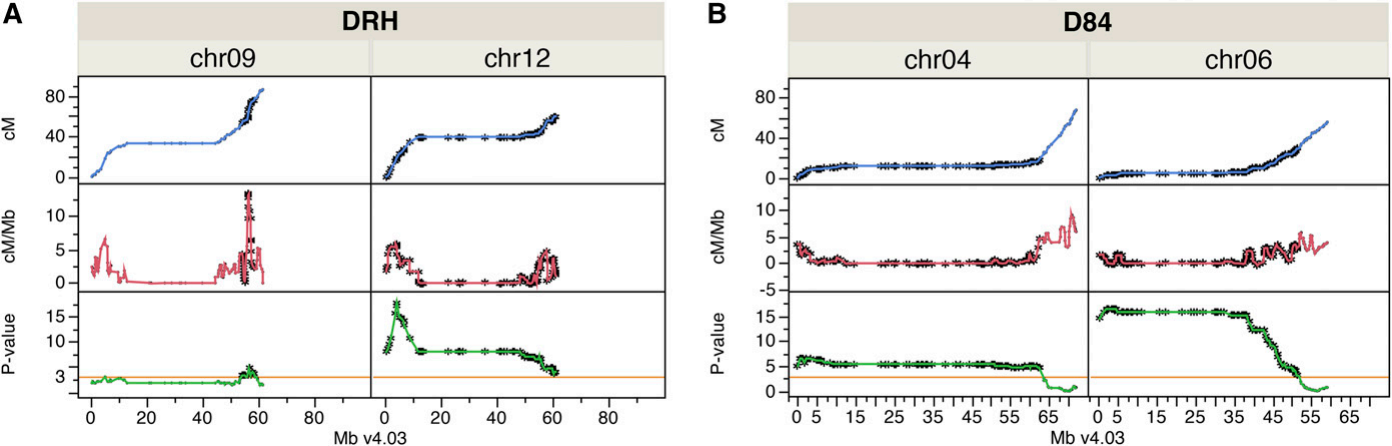
Distribution of recombination rates along chromosomes with distorted segregation regions. (A) Chromosomes (chr) 9 and 12 for the DRH population, (B) chromosomes 4 and 6 for D84. For each chromosome square, the upper panel is the Marey map, the middle panel is the recombination rate (cM/Mb), and the lower panel is the significance of distorted segregation reported as the minus logarithm of the chi-square test *P*-value (*P*-value), plotted against physical position in Mb based on potato genome assembly version 4.03 (Mb v4.03). The 0.1% threshold of significance used to define distorted segregation corresponds to the orange line of 3. Black stars highlight loci with distorted segregation.

Comparison between genetic and physical maps was used to estimate genome-wide variation of recombination rates. Initially, we analyzed the averages of genome-wide and chromosome recombination rates for each population (Table 6). The average recombination rates were 1.1, 0.9, and 1.1 cM/Mb for DRH, D84, and MSX902, respectively. The average recombination rate per chromosome varied among and within populations, ranging from 0.9–1.5, 0.7–1.2, and 0.9–1.7 cM/Mb for DRH, D84, and MSX902, respectively. In general, D84 had the lowest recombination rates at the genome and chromosome levels. Chromosomes 2 and 11 had the greatest recombination rates for most of the populations, followed by chromosome 5 for DRH and MSX902. The recombination rates along chromosomes increased toward the telomeres, and decreased toward the centromere regions, as expected. SDRs were indiscriminately distributed along the centromere and arms of the chromosomes with variable recombination rates (Figure 2, Figure S4, Figure S5, Figure S6, and Figure S7). Regression analysis between recombination rates and minus logarithm of the chi-square test *P*-value of segregating genotypes per locus was performed for chromosomes with SDRs. Loci with distorted segregation were defined by values greater than three (equivalent to α less than 0.001). There was significant (*) positive correlation between greater recombination rates and the most distorted loci on chromosome 9 (*n* = 166, *r^2^* = 0.25, *P* < 0.0001*) and chromosome 12 (*n* = 121, *r*^2^ = 0.41, *P* < 0.001*) in DRH (Figure 2A). On the other hand, lower recombination rates correlated with greater levels of distorted segregation were common for chromosomes with SDRs in D84 (*n* = 267, *r*^2^ = 0.11, *P* < 0.0001*; *n* = 225, *r*^2^ = 0.38, *P* < 0.0001*; *n* = 154, *r*^2^ = 0.8, *P* < 0.0001*; *n* = 205, *r*^2^ = 0.73, *P* < 0.0001*; *n* = 241, *r*^2^ = 0.7, *P* < 0.0001*; *n* = 171, *r*^2^ = 0.51, *P* < 0.0001* for chromosomes 2, 3, 4, 6, 7, and 8, respectively). For MSX902, there was no correlation between recombination rates and distorted segregation on chromosomes 2 and 9 for the 84SD22 parental map (*n* = 164, *r*^2^ = 0.03, *P* = 0.04, and *n* = 88, *r*^2^ = 0.01, *P* = 0.42). Similar results were found for *Ber*83 on chromosomes 1, 2, and 7 (*n* = 95, *r*^2^ = 0.01, *P* = 0.32; *n* = 53, *r*^2^ = 0.001, *P* = 0.83; and *n* = 83, *r*^2^ = 0.02, *P* = 0.2). However, a positive correlation between recombination rates and distorted segregation was detected on chromosome 12 (*n* = 63, *r*^2^ = 0.28, *P* < 0.0001*) of the *Ber*83 map.

**Table 6:** Average of genome-wide and chromosome recombination rates in cM/Mb for three diploid populations

Chromosome	DRH	D84	MSX902 (Combined)	P1 MSX902 (84SD22)	P2 MSX902 (*Ber*83)
chr01	1.1	0.7	0.9	0.9	0.9
chr02	1.4	1.2	1.5	1.5	1.6
chr03	1.1	0.8	0.9	1.0	0.7
chr04	1.1	0.9	1.0	1.1	0.8
chr05	1.4	1.0	1.2	1.1	1.3
chr06	0.9	0.9	1.0	1.1	0.8
chr07	0.9	0.8	1.1	1.2	1.1
chr08	1.0	1.0	1.0	0.9	1.2
chr09	1.4	0.9	1.0	0.9	1.0
chr10	1.0	0.9	1.1	1.2	1.0
chr11	1.5	0.9	1.7	1.6	1.7
chr12	1.0	0.9	1.1	1.3	0.9
Genome-wide	1.1	0.9	1.1	1.1	1.0

The spatial and temporal context where hybridization occurs modulates the outcome, breakdown, or strengthening of barriers to gene exchange (Abbott *et al.* 2013). A balance between selection and recombination in the framework of the cline theory explains the dynamics to stabilize or increase reproductive barriers to gene exchange. In the context of genome divergence and recombination levels *vs.* the development of hybridizations barriers, we found that interspecific hybridity of parental clones, and androgenic rather than gynogenic gamete formation, were associated with lower recombination rates in this study. In general, there were smaller genetic map lengths from male than female parents and from inter *vs.* intraspecific crosses. The 813.2 cM DRH map, with 756 recombination events and an average of 7.9 per plant (*S. tuberosum* Group Phureja and Tuberosum cross), was larger than D84 (637.9 cM) and *Ber*83 (730.3 cM) with 810 and 916 recombination events and an average of 6.2 and 7.1 per plant, respectively. However, 84SD22 with 808.1 cM showed greater recombination frequencies (1015 recombination events, 7.9 per plant) when acting as female parent in MSX902. In the D84 and MSX902 populations, the hybrid parents contained germplasm from *S. tuberosum* Groups Phureja and Tuberosum, *S. chacoense*, and *S. berthaultii*, compared to the *S. tuberosum* Groups Phureja and Tuberosum background of DRH. Similar results were reported by Gebhardt *et al.* (1991), *i.e.*, smaller genetic maps associated with lower recombination frequency in parental clones with interspecific hybridity compared to parents with intraspecific genetic background.

Checking the recombination rates in the regions with distorted segregation in the progeny, we found six and four SDRs in D84 and MSX902 interspecific crosses in the male parents (84SD22 and *Ber*83), compared to two in 84SD22 as the female parent of MSX902 or two in DRH, a more intraspecific cross. The SDRs in the 84SD22 and *Ber*83 male maps were mainly associated with lower local recombination rates. However, for chromosome 9 in DRH and 12 in DRH and MSX902, the SDRs from the male parent were located in regions with greater recombination rates. Interspecific male parents with lower total recombination rates and high levels of distorted segregation have been reported (Bonierbale *et al.* 1988; Gebhardt *et al.* 1991). Likewise, greater levels of distorted segregation from the male parent were found in an intraspecific cross (Jacobs *et al.* 1995), and lower recombination rates combined with greater percentages of distorted segregation of a *S. tuberosum* clone when used as a male rather than female parent (Kreike and Stiekema 1997). Therefore, there is a clear pattern where greater levels of genome divergence in the parent genome produced lower proportions of recombination events, and those regions with lower recombination rates were the ones that suffered distorted segregation.

### Conclusions

Given the differences in the occurrence of distorted segregation among the three diploid populations in our study, even when sharing a common parent, it seems likely that segregation distortion in a highly heterozygous crop such as potato will be population specific, reflecting the diverse genetic load within selected parents. Interspecific hybridity of parental clones and sex-specific recombination rates were factors associated with SDR. The presence of meiotic mutations, deleterious alleles producing male gamete abortion or sterility, gametic competition, the self-incompatibility locus, the *S-locus inhibitor* gene, and interspecific incongruity were all considered to be potentially associated with distorted segregation in this study. The SNP genotyping platform used in this study was important not only to study segregation distortion, but also to compare three high-density colinear genetic maps. We found that genotype errors corresponded to the most limiting factor to obtain high quality maps rather than distorted segregation.

## Supplementary Material

Supplemental Material
