# Supplementary material for: Comparative Analysis of Regions with Distorted Segregation in Three Diploid Populations of Potato
Source: G3 (Bethesda). 2016 Jun 23;6(8):2617–28. doi: 10.1534/g3.116.030031 (PMC4978915; doi:10.1534/g3.116.030031)
Supplement: Supplemental Material [file supp_6_8_2617__index.html]

Comparative Analysis of Regions with Distorted Segregation in Three Diploid Populations of Potato — Supplemental Material 

# Comparative Analysis of Regions with Distorted Segregation in Three Diploid Populations of Potato

## Supplemental Material for Manrique-Carpintero *et al.*, 2016

**Files in this Data Supplement:**

- Figure S1 - Pedigree scheme of *Ber83*. A dihaploid of *Solanum tuberosum* Group Tuberosum was cross with a *Solanum chacoense* clone to generate the 84SD22 hybrid. 84SD22 was crossed to a *S. tuberosum* Group Phureja clone to generate MSA133-57. Finally MSA133-57 was crossed to *Solanum berthaultii* PI498104 to obtain the hybrid *Ber83*. (.pdf, 65 KB)
- Figure S2 - Comparative map of DRH, D84 and MSX902 genetic maps. (.pdf, 5324 KB)
- Figure S3 - Distribution of segregation ratios of parental haplotype combinations along the genetic linkage map (cM) for MSX902 population. (.pdf, 409 KB)
- Figure S4 - Distribution of recombination rates along chromosomes with distorted segregation regions for DRH population. (.pdf, 475 KB)
- Figure S5 - Distribution of recombination rates along chromosomes with distorted segregation regions for D84 population. (.pdf, 395 KB)
- Figure S6 - Distribution of recombination rates along chromosomes with distorted segregation regions for 84SD22 female parent P1 of MSX902 population. (.pdf, 312 KB)
- Figure S7 - Distribution of recombination rates along chromosomes with distorted segregation regions for *Ber83* male parent P2 of MSX902 population. For each chromosome (chr), in the upper panel is the Marey map, the middle panel is the recombination rate (cM/Mb), and the lower panel is the significance of distorted segregation reported as the minus logarithm of chi square test P-value (P-value), plotted against physical position in Mb based on potato genome assembly version 4.03 (Mb v4.03). The 0.1% threshold of significance used to define distorted segregation corresponds to orange line of 3. Black stars highlight loci with distorted segregation. (.pdf, 231 KB)
- Table S1 - Number of loci with distorted and expected segregation based on a Chi-square test using four thresholds of significance (5%, 1%, 0.1% and 0.001%) for DRH population. (.xlsx, 45 KB)
- Table S2 - Number of loci with distorted and expected segregation based on a Chi-square test using four thresholds of significance (5%, 1%, 0.1% and 0.001%) for D84 population. (.xlsx, 42 KB)
- Table S3 - Number of loci with distorted and expected segregation based on a Chi-square test using four thresholds of significance (5%, 1%, 0.1% and 0.001%) for MSX902 population. (.xlsx, 47 KB)
- Table S4 - Number of hkxhk loci with distorted and expected segregation based on a Chi-square test using four thresholds of significance (5%, 1%, 0.1% and 0.001%) for MSX902 population. (.xlsx, 43 KB)
- Table S5 - Number of lmxll loci with distorted and expected segregation based on a Chi-square test using four thresholds of significance (5%, 1%, 0.1% and 0.001%) for 84SD22 parent of MSX902 population. (.xlsx, 43 KB)
- Table S6 - Number of nnxnp loci with distorted and expected segregation based on a Chi-square test using four thresholds of significance (5%, 1%, 0.1% and 0.001%) for Ber83 parent of MSX902 population. (.xlsx, 44 KB)
- Table S7 - Significant nonrandom association between unlinked loci on DRH cross. (.xlsx, 44 KB)
- Table S8 - Significant nonrandom association between unlinked loci on D84 cross. (.xlsx, 57 KB)
- Table S9 - Significant nonrandom association between unlinked loci on MSX902 cross. (.xlsx, 96 KB)
