# Supplementary material for: Comparative Analysis of Regions with Distorted Segregation in Three Diploid Populations of Potato
Source: G3 (Bethesda). 2016 Jun 23;6(8):2617–28. doi: 10.1534/g3.116.030031 (PMC4978915; doi:10.1534/g3.116.030031)
Supplement: Supplemental Material [file supp_g3.116.030031_FigureS1.pdf]

US-W3304 Merrimack

Dihaploid of *S. tuberosum* × 1-24-1 *S. chacoense*

↓

84SD22 × 84S10 *S. phureja*

↓

MSA133-57 × PI498104 *S. berthaultii*

↓

Ber83

Fig. S1. Pedigree scheme of Ber83. A dihaploid of Solanum tuberosum Group Tuberosum was cross with a Solanum chacoense clone to generate the 84SD22 hybrid. 84SD22 was crossed to a S. tuberosum Group Phureja clone to generate MSA133-57. Finally MSA133-57 was crossed to Solanum berthaultii PI498104 to obtain the hybrid Ber83.
