# Supplementary material for: Comparative Analysis of Regions with Distorted Segregation in Three Diploid Populations of Potato
Source: G3 (Bethesda). 2016 Jun 23;6(8):2617–28. doi: 10.1534/g3.116.030031 (PMC4978915; doi:10.1534/g3.116.030031)
Supplement: Supplemental Material [file supp_g3.116.030031_FigureS2.pdf]

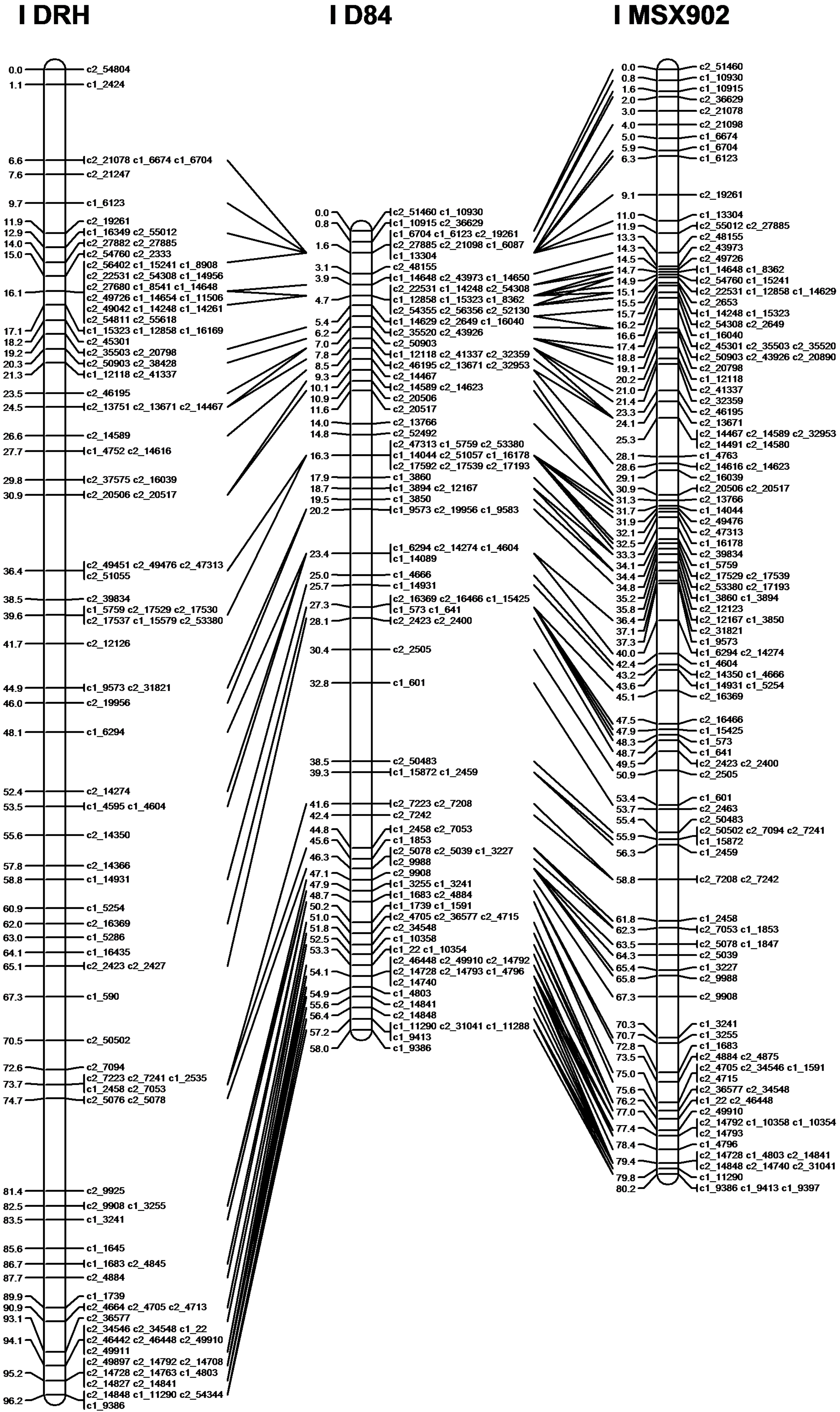
I DRH
I D84
I MSX902
0.0 c2_54804
1.1 c1_2424
6.6 c2_21078 c1_6674 c1_6704
7.6 c2_21247
9.7 c1_6123
11.9 c2_19261
12.9 c1_16349 c2_55012
14.0 c2_27882 c2_27885
15.0 c2_54760 c2_2333
16.1 c2_56402 c1_15241 c1_8908 c2_22531 c2_54308 c1_14956 c2_27680 c1_8541 c1_14648 c2_49726 c1_14654 c1_11506 c2_49042 c1_14248 c1_14261 c2_54811 c2_55618
17.1 c1_15323 c1_12858 c1_16169
18.2 c2_45301
19.2 c2_35503 c2_20798
20.3 c2_50903 c2_38428
21.3 c1_12118 c2_41337
23.5 c2_46195
24.5 c2_13751 c2_13671 c2_14467
26.6 c2_14589
27.7 c1_4752 c2_14616
29.8 c2_37575 c2_16039
30.9 c2_20506 c2_20517
36.4 c2_49451 c2_49476 c2_47313 c2_51055
38.5 c2_39834
39.6 c1_5759 c2_17529 c2_17530 c2_17537 c1_15579 c2_53380
41.7 c2_12126
44.9 c1_9573 c2_31821
46.0 c2_19956
48.1 c1_6294
52.4 c2_14274
53.5 c1_4595 c1_4604
55.6 c2_14350
57.8 c2_14366
58.8 c1_14931
60.9 c1_5254
62.0 c2_16369
63.0 c1_5286
64.1 c1_16435
65.1 c2_2423 c2_2427
67.3 c1_590
70.5 c2_50502
72.6 c2_7094
73.7 c2_7223 c2_7241 c1_2535 c1_2458 c2_7053
74.7 c2_5076 c2_5078
81.4 c2_9925
82.5 c2_9908 c1_3255
83.5 c1_3241
85.6 c1_1645
86.7 c1_1683 c2_4845
87.7 c2_4884
89.9 c1_1739
90.9 c2_4664 c2_4705 c2_4713
93.1 c2_36577
94.1 c2_34546 c2_34548 c1_22 c2_46442 c2_46448 c2_49910 c2_49911
95.2 c2_49897 c2_14792 c2_14708 c2_14728 c2_14763 c1_4803 c2_14827 c2_14841
96.2 c2_14848 c1_11290 c2_54344 c1_9386
0.0 c2_51460 c1_10930
0.8 c1_10915 c2_36629
1.6 c1_6704 c1_6123 c2_19261 c2_27885 c2_21098 c1_6087 c1_13304
3.1 c2_48155
3.9 c1_14648 c2_43973 c1_14650
4.7 c2_22531 c1_14248 c2_54308 c1_12858 c1_15323 c1_8362 c2_54355 c2_56356 c2_52130
5.4 c1_14629 c2_2649 c1_16040
6.2 c2_35520 c2_43926
7.0 c2_50903
7.8 c1_12118 c2_41337 c2_32359
8.5 c2_46195 c2_13671 c2_32953
9.3 c2_14467
10.1 c2_14589 c2_14623
10.9 c2_20506
11.6 c2_20517
14.0 c2_13766
14.8 c2_52492
16.3 c2_47313 c1_5759 c2_53380 c1_14044 c2_51057 c1_16178 c2_17592 c2_17539 c2_17193
17.9 c1_3860
18.7 c1_3894 c2_12167
19.5 c1_3850
20.2 c1_9573 c2_19956 c1_9583
23.4 c1_6294 c2_14274 c1_4604 c1_14089
25.0 c1_4666
25.7 c1_14931
27.3 c2_16369 c2_16466 c1_15425 c1_573 c1_641
28.1 c2_2423 c2_2400
30.4 c2_2505
32.8 c1_601
38.5 c2_50483
39.3 c1_15872 c1_2459
41.6 c2_7223 c2_7208
42.4 c2_7242
44.8 c1_2458 c2_7053
45.6 c1_1853
46.3 c2_5078 c2_5039 c1_3227 c2_9988
47.1 c2_9908
47.9 c1_3255 c1_3241
48.7 c1_1683 c2_4884
50.2 c1_1739 c1_1591
51.0 c2_4705 c2_36577 c2_4715
51.8 c2_34548
52.5 c1_10358
53.3 c1_22 c1_10354
54.1 c2_46448 c2_49910 c2_14792 c2_14728 c2_14793 c1_4796 c2_14740
54.9 c1_4803
55.6 c2_14841
56.4 c2_14848
57.2 c1_11290 c2_31041 c1_11288 c1_9413
58.0 c1_9386
0.0 c2_51460
0.8 c1_10930
1.6 c1_10915
2.0 c2_36629
3.0 c2_21078
4.0 c2_21098
5.0 c1_6674
5.9 c1_6704
6.3 c1_6123
9.1 c2_19261
11.0 c1_13304
11.9 c2_55012 c2_27885
13.3 c2_48155
14.3 c2_43973
14.5 c2_49726
14.7 c1_14648 c1_8362
14.9 c2_54760 c1_15241
15.1 c2_22531 c1_12858 c1_14629
15.5 c2_2653
15.7 c1_14248 c1_15323
16.2 c2_54308 c2_2649
16.6 c1_16040
17.4 c2_45301 c2_35503 c2_35520
18.8 c2_50903 c2_43926 c2_20890
19.1 c2_20798
20.2 c1_12118
21.0 c2_41337
21.4 c2_32359
23.3 c2_46195
24.1 c2_13671
25.3 c2_14467 c2_14589 c2_32953 c2_14491 c2_14580
28.1 c1_4763
28.6 c2_14616 c2_14623
29.1 c2_16039
30.9 c2_20506 c2_20517
31.3 c2_13766
31.7 c1_14044
31.9 c2_49476
32.1 c2_47313
32.5 c1_16178
33.3 c2_39834
34.1 c1_5759
34.4 c2_17529 c2_17539
34.8 c2_53380 c2_17193
35.2 c1_3860 c1_3894
35.8 c2_12123
36.4 c2_12167 c1_3850
37.1 c2_31821
37.3 c1_9573
40.0 c1_6294 c2_14274
42.4 c1_4604
43.2 c2_14350 c1_4666
43.6 c1_14931 c1_5254
45.1 c2_16369
47.5 c2_16466
47.9 c1_15425
48.3 c1_573
48.7 c1_641
49.5 c2_2423 c2_2400
50.9 c2_2505
53.4 c1_601
53.7 c2_2463
55.4 c2_50483
55.9 c2_50502 c2_7094 c2_7241 c1_15872
56.3 c1_2459
58.8 c2_7208 c2_7242
61.8 c1_2458
62.3 c2_7053 c1_1853
63.5 c2_5078 c1_1847
64.3 c2_5039
65.4 c1_3227
65.8 c2_9988
67.3 c2_9908
70.3 c1_3241
70.7 c1_3255
72.8 c1_1683
73.5 c2_4884 c2_4875
75.0 c2_4705 c2_34546 c1_1591 c2_4715
75.6 c2_36577 c2_34548
76.2 c1_22 c2_46448
77.0 c2_49910
77.4 c2_14792 c1_10358 c1_10354 c2_14793
78.4 c1_4796
79.4 c2_14728 c1_4803 c2_14841 c2_14848 c2_14740 c2_31041
79.8 c1_11290
80.2 c1_9386 c1_9413 c1_9397

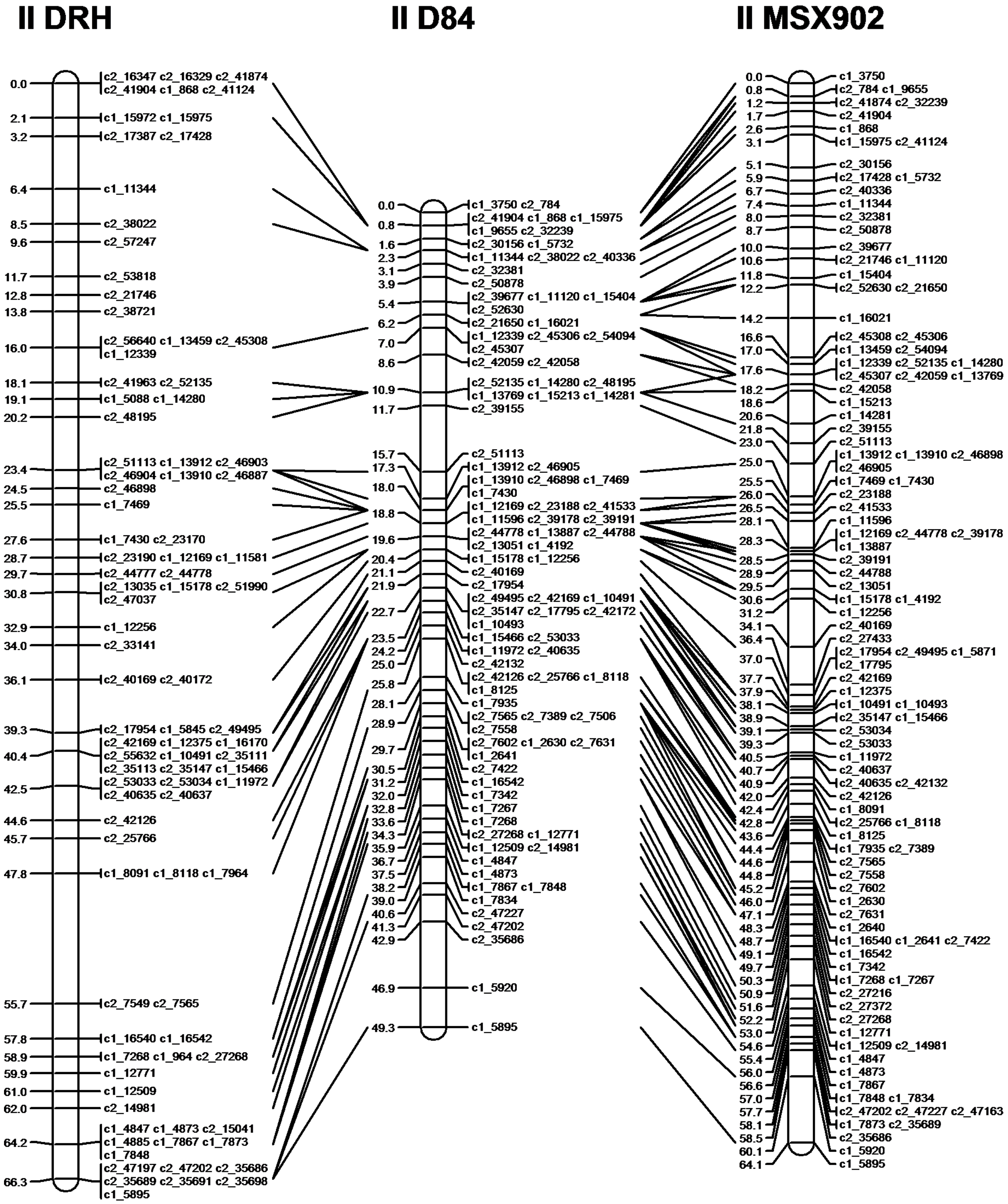

II DRH
II D84
II MSX902

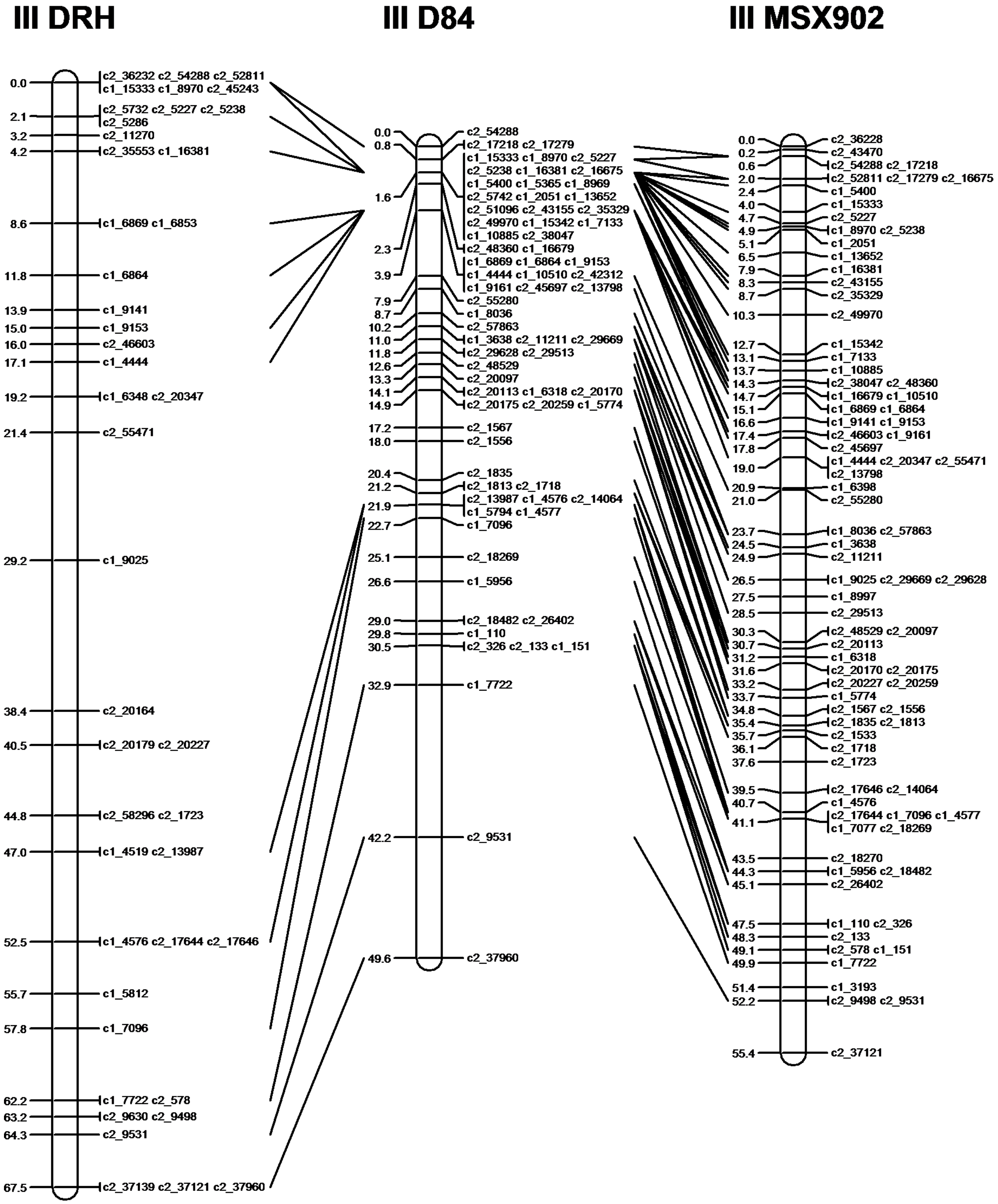
III DRH
0.0 c2_36232 c2_54288 c2_52811
c1_15333 c1_8970 c2_45243
2.1 c2_5732 c2_5227 c2_5238
c2_5286
3.2 c2_11270
4.2 c2_35553 c1_16381
8.6 c1_6869 c1_6853
11.8 c1_6864
13.9 c1_9141
15.0 c1_9153
16.0 c2_46603
17.1 c1_4444
19.2 c1_6348 c2_20347
21.4 c2_55471
29.2 c1_9025
38.4 c2_20164
40.5 c2_20179 c2_20227
44.8 c2_58296 c2_1723
47.0 c1_4519 c2_13987
52.5 c1_4576 c2_17644 c2_17646
55.7 c1_5812
57.8 c1_7096
62.2 c1_7722 c2_578
63.2 c2_9630 c2_9498
64.3 c2_9531
67.5 c2_37139 c2_37121 c2_37960
III D84
0.0 c2_54288
0.8 c2_17218 c2_17279
c1_15333 c1_8970 c2_5227
c2_5238 c1_16381 c2_16675
c1_5400 c1_5365 c1_8969
1.6 c2_5742 c1_2051 c1_13652
c2_51096 c2_43155 c2_35329
c2_49970 c1_15342 c1_7133
c1_10885 c2_38047
2.3 c2_48360 c1_16679
c1_6869 c1_6864 c1_9153
3.9 c1_4444 c1_10510 c2_42312
c1_9161 c2_45697 c2_13798
7.9 c2_55280
8.7 c1_8036
10.2 c2_57863
11.0 c1_3638 c2_11211 c2_29669
11.8 c2_29628 c2_29513
12.6 c2_48529
13.3 c2_20097
14.1 c2_20113 c1_6318 c2_20170
14.9 c2_20175 c2_20259 c1_5774
17.2 c2_1567
18.0 c2_1556
20.4 c2_1835
21.2 c2_1813 c2_1718
21.9 c2_13987 c1_4576 c2_14064
c1_5794 c1_4577
22.7 c1_7096
25.1 c2_18269
26.6 c1_5956
29.0 c2_18482 c2_26402
29.8 c1_110
30.5 c2_326 c2_133 c1_151
32.9 c1_7722
42.2 c2_9531
49.6 c2_37960
III MSX902
0.0 c2_36228
0.2 c2_43470
0.6 c2_54288 c2_17218
2.0 c2_52811 c2_17279 c2_16675
2.4 c1_5400
4.0 c1_15333
4.7 c2_5227
4.9 c1_8970 c2_5238
5.1 c1_2051
6.5 c1_13652
7.9 c1_16381
8.3 c2_43155
8.7 c2_35329
10.3 c2_49970
12.7 c1_15342
13.1 c1_7133
13.7 c1_10885
14.3 c2_38047 c2_48360
14.7 c1_16679 c1_10510
15.1 c1_6869 c1_6864
16.6 c1_9141 c1_9153
17.4 c2_46603 c1_9161
17.8 c2_45697
19.0 c1_4444 c2_20347 c2_55471
c2_13798
20.9 c1_6398
21.0 c2_55280
23.7 c1_8036 c2_57863
24.5 c1_3638
24.9 c2_11211
26.5 c1_9025 c2_29669 c2_29628
27.5 c1_8997
28.5 c2_29513
30.3 c2_48529 c2_20097
30.7 c2_20113
31.2 c1_6318
31.6 c2_20170 c2_20175
33.2 c2_20227 c2_20259
33.7 c1_5774
34.8 c2_1567 c2_1556
35.4 c2_1835 c2_1813
35.7 c2_1533
36.1 c2_1718
37.6 c2_1723
39.5 c2_17646 c2_14064
40.7 c1_4576
41.1 c2_17644 c1_7096 c1_4577
c1_7077 c2_18269
43.5 c2_18270
44.3 c1_5956 c2_18482
45.1 c2_26402
47.5 c1_110 c2_326
48.3 c2_133
49.1 c2_578 c1_151
49.9 c1_7722
51.4 c1_3193
52.2 c2_9498 c2_9531
55.4 c2_37121

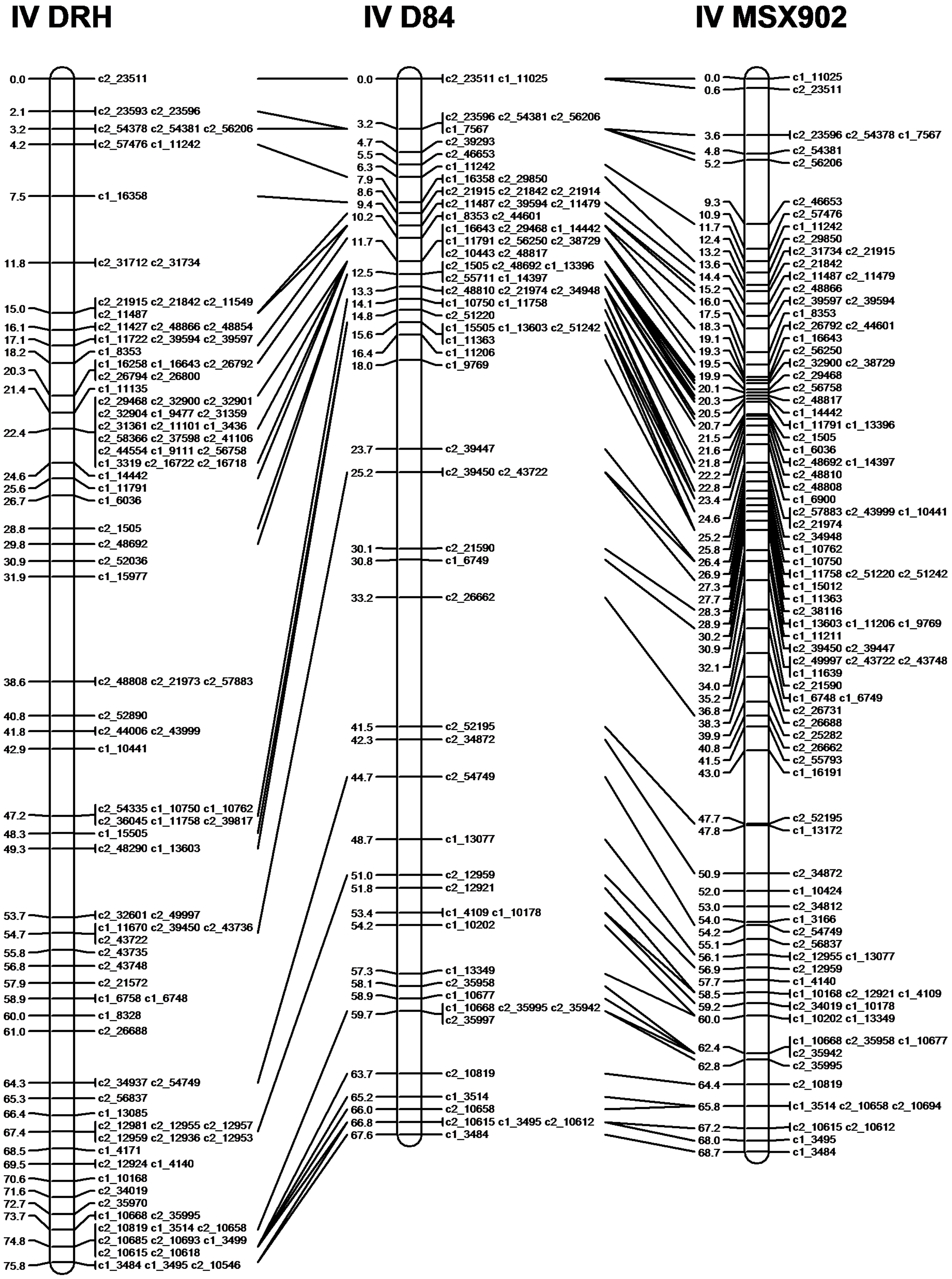
IV DRH
0.0 c2_23511
2.1 c2_23593 c2_23596
3.2 c2_54378 c2_54381 c2_56206
4.2 c2_57476 c1_11242
7.5 c1_16358
11.8 c2_31712 c2_31734
15.0 c2_21915 c2_21842 c2_11549 c2_11487
16.1 c2_11427 c2_48866 c2_48854
17.1 c1_11722 c2_39594 c2_39597
18.2 c1_8353
20.3 c1_16258 c1_16643 c2_26792 c2_26794 c2_26800
21.4 c1_11135
22.4 c2_29468 c2_32900 c2_32901 c2_32904 c1_9477 c2_31359 c2_31361 c2_11101 c1_3436 c2_58366 c2_37598 c2_41106 c2_44554 c1_9111 c2_56758 c1_3319 c2_16722 c2_16718
24.6 c1_14442
25.6 c1_11791
26.7 c1_6036
28.8 c2_1505
29.8 c2_48692
30.9 c2_52036
31.9 c1_15977
38.6 c2_48808 c2_21973 c2_57883
40.8 c2_52890
41.8 c2_44006 c2_43999
42.9 c1_10441
47.2 c2_54335 c1_10750 c1_10762 c2_36045 c1_11758 c2_39817
48.3 c1_15505
49.3 c2_48290 c1_13603
53.7 c2_32601 c2_49997
54.7 c1_11670 c2_39450 c2_43736 c2_43722
55.8 c2_43735
56.8 c2_43748
57.9 c2_21572
58.9 c1_6758 c1_6748
60.0 c1_8328
61.0 c2_26688
64.3 c2_34937 c2_54749
65.3 c2_56837
66.4 c1_13085
67.4 c2_12981 c2_12955 c2_12957 c2_12959 c2_12936 c2_12953
68.5 c1_4171
69.5 c2_12924 c1_4140
70.6 c1_10168
71.6 c2_34019
72.7 c2_35970
73.7 c1_10668 c2_35995
74.8 c2_10819 c1_3514 c2_10658 c2_10685 c2_10693 c1_3499 c2_10615 c2_10618
75.8 c1_3484 c1_3495 c2_10546
IV D84
0.0 c2_23511 c1_11025
3.2 c2_23596 c2_54381 c2_56206 c1_7567
4.7 c2_39293
5.5 c2_46653
6.3 c1_11242
7.9 c1_16358 c2_29850
8.6 c2_21915 c2_21842 c2_21914
9.4 c2_11487 c2_39594 c2_11479
10.2 c1_8353 c2_44601
11.7 c1_16643 c2_29468 c1_14442 c1_11791 c2_56250 c2_38729 c2_10443 c2_48817
12.5 c2_1505 c2_48692 c1_13396 c2_55711 c1_14397
13.3 c2_48810 c2_21974 c2_34948
14.1 c1_10750 c1_11758
14.8 c2_51220
15.6 c1_15505 c1_13603 c2_51242 c1_11363
16.4 c1_11206
18.0 c1_9769
23.7 c2_39447
25.2 c2_39450 c2_43722
30.1 c2_21590
30.8 c1_6749
33.2 c2_26662
41.5 c2_52195
42.3 c2_34872
44.7 c2_54749
48.7 c1_13077
51.0 c2_12959
51.8 c2_12921
53.4 c1_4109 c1_10178
54.2 c1_10202
57.3 c1_13349
58.1 c2_35958
58.9 c1_10677
59.7 c1_10668 c2_35995 c2_35942 c2_35997
63.7 c2_10819
65.2 c1_3514
66.0 c2_10658
66.8 c2_10615 c1_3495 c2_10612
67.6 c1_3484
IV MSX902
0.0 c1_11025
0.6 c2_23511
3.6 c2_23596 c2_54378 c1_7567
4.8 c2_54381
5.2 c2_56206
9.3 c2_46653
10.9 c2_57476
11.7 c1_11242
12.4 c2_29850
13.2 c2_31734 c2_21915
13.6 c2_21842
14.4 c2_11487 c2_11479
15.2 c2_48866
16.0 c2_39597 c2_39594
17.5 c1_8353
18.3 c2_26792 c2_44601
19.1 c1_16643
19.3 c2_56250
19.5 c2_32900 c2_38729
19.9 c2_29468
20.1 c2_56758
20.3 c2_48817
20.5 c1_14442
20.7 c1_11791 c1_13396
21.5 c2_1505
21.6 c1_6036
21.8 c2_48692 c1_14397
22.2 c2_48810
22.8 c2_48808
23.4 c1_6900
24.6 c2_57883 c2_43999 c1_10441 c2_21974
25.2 c2_34948
25.8 c1_10762
26.4 c1_10750
26.9 c1_11758 c2_51220 c2_51242
27.3 c1_15012
27.7 c1_11363
28.3 c2_38116
28.9 c1_13603 c1_11206 c1_9769
30.2 c1_11211
30.9 c2_39450 c2_39447
32.1 c2_49997 c2_43722 c2_43748 c1_11639
34.0 c2_21590
35.2 c1_6748 c1_6749
36.8 c2_26731
38.3 c2_26688
39.9 c2_25282
40.8 c2_26662
41.5 c2_55793
43.0 c1_16191
47.7 c2_52195
47.8 c1_13172
50.9 c2_34872
52.0 c1_10424
53.0 c2_34812
54.0 c1_3166
54.2 c2_54749
55.1 c2_56837
56.1 c2_12955 c1_13077
56.9 c2_12959
57.7 c1_4140
58.5 c1_10168 c2_12921 c1_4109
59.2 c2_34019 c1_10178
60.0 c1_10202 c1_13349
62.4 c1_10668 c2_35958 c1_10677 c2_35942
62.8 c2_35995
64.4 c2_10819
65.8 c1_3514 c2_10658 c2_10694
67.2 c2_10615 c2_10612
68.0 c1_3495
68.7 c1_3484

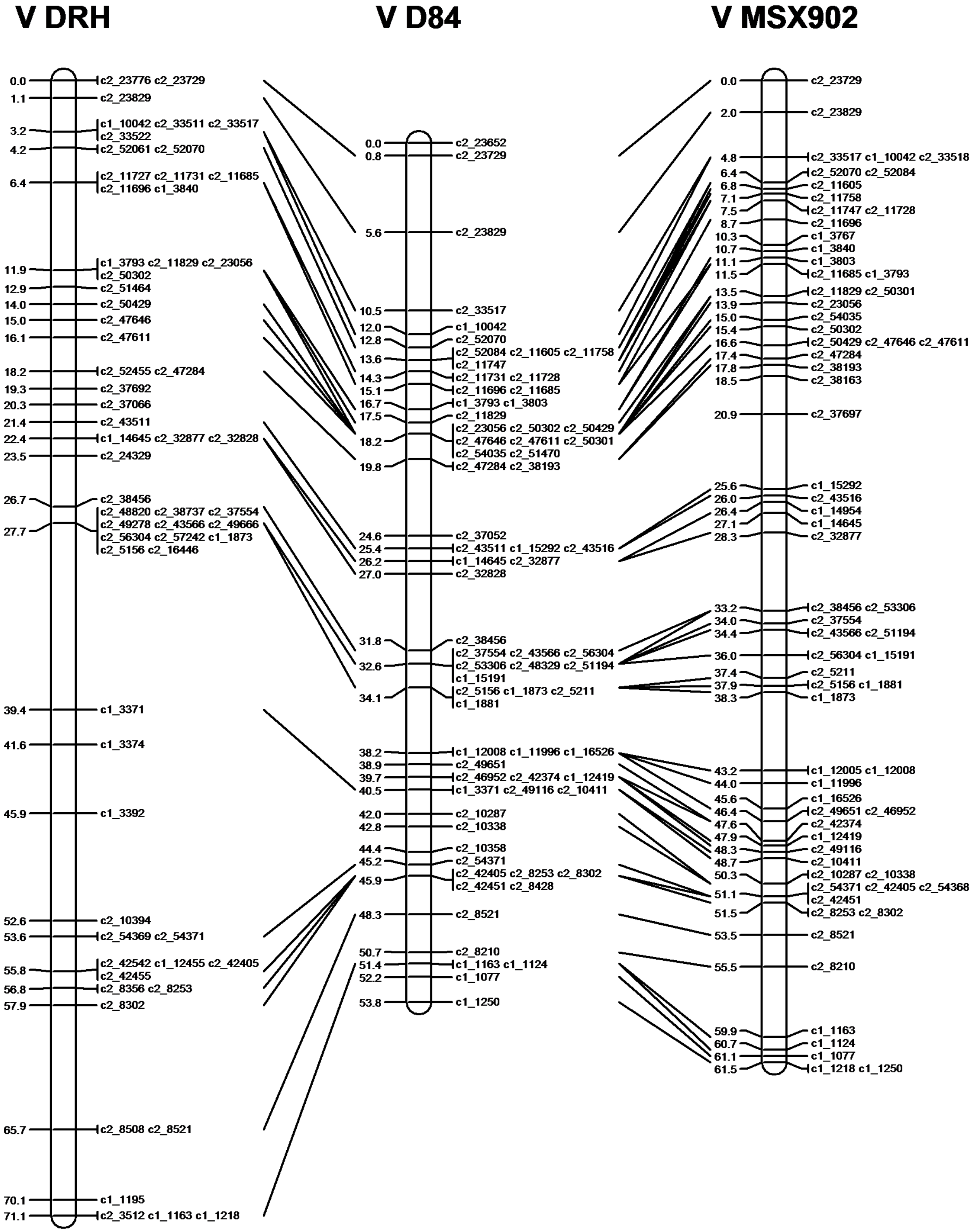

V DRH
0.0 c2_23776 c2_23729
1.1 c2_23829
3.2 c1_10042 c2_33511 c2_33517 c2_33522
4.2 c2_52061 c2_52070
6.4 c2_11727 c2_11731 c2_11685 c2_11696 c1_3840
11.9 c1_3793 c2_11829 c2_23056 c2_50302
12.9 c2_51464
14.0 c2_50429
15.0 c2_47646
16.1 c2_47611
18.2 c2_52455 c2_47284
19.3 c2_37692
20.3 c2_37066
21.4 c2_43511
22.4 c1_14645 c2_32877 c2_32828
23.5 c2_24329
26.7 c2_38456
27.7 c2_48820 c2_38737 c2_37554 c2_49278 c2_43566 c2_49666 c2_56304 c2_57242 c1_1873 c2_5156 c2_16446
39.4 c1_3371
41.6 c1_3374
45.9 c1_3392
52.6 c2_10394
53.6 c2_54369 c2_54371
55.8 c2_42542 c1_12455 c2_42405 c2_42455
56.8 c2_8356 c2_8253
57.9 c2_8302
65.7 c2_8508 c2_8521
70.1 c1_1195
71.1 c2_3512 c1_1163 c1_1218
V D84
0.0 c2_23652
0.8 c2_23729
5.6 c2_23829
10.5 c2_33517
12.0 c1_10042
12.8 c2_52070
13.6 c2_52084 c2_11605 c2_11758 c2_11747
14.3 c2_11731 c2_11728
15.1 c2_11696 c2_11685
16.7 c1_3793 c1_3803
17.5 c2_11829
18.2 c2_23056 c2_50302 c2_50429 c2_47646 c2_47611 c2_50301 c2_54035 c2_51470
19.8 c2_47284 c2_38193
24.6 c2_37052
25.4 c2_43511 c1_15292 c2_43516
26.2 c1_14645 c2_32877
27.0 c2_32828
31.8 c2_38456
32.6 c2_37554 c2_43566 c2_56304 c2_53306 c2_48329 c2_51194 c1_15191
34.1 c2_5156 c1_1873 c2_5211 c1_1881
38.2 c1_12008 c1_11996 c1_16526
38.9 c2_49651
39.7 c2_46952 c2_42374 c1_12419
40.5 c1_3371 c2_49116 c2_10411
42.0 c2_10287
42.8 c2_10338
44.4 c2_10358
45.2 c2_54371
45.9 c2_42405 c2_8253 c2_8302 c2_42451 c2_8428
48.3 c2_8521
50.7 c2_8210
51.4 c1_1163 c1_1124
52.2 c1_1077
53.8 c1_1250
V MSX902
0.0 c2_23729
2.0 c2_23829
4.8 c2_33517 c1_10042 c2_33518
6.4 c2_52070 c2_52084
6.8 c2_11605
7.1 c2_11758
7.5 c2_11747 c2_11728
8.7 c2_11696
10.3 c1_3767
10.7 c1_3840
11.1 c1_3803
11.5 c2_11685 c1_3793
13.5 c2_11829 c2_50301
13.9 c2_23056
15.0 c2_54035
15.4 c2_50302
16.6 c2_50429 c2_47646 c2_47611
17.4 c2_47284
17.8 c2_38193
18.5 c2_38163
20.9 c2_37697
25.6 c1_15292
26.0 c2_43516
26.4 c1_14954
27.1 c1_14645
28.3 c2_32877
33.2 c2_38456 c2_53306
34.0 c2_37554
34.4 c2_43566 c2_51194
36.0 c2_56304 c1_15191
37.4 c2_5211
37.9 c2_5156 c1_1881
38.3 c1_1873
43.2 c1_12005 c1_12008
44.0 c1_11996
45.6 c1_16526
46.4 c2_49651 c2_46952
47.6 c2_42374
47.9 c1_12419
48.3 c2_49116
48.7 c2_10411
50.3 c2_10287 c2_10338
51.1 c2_54371 c2_42405 c2_54368 c2_42451
51.5 c2_8253 c2_8302
53.5 c2_8521
55.5 c2_8210
59.9 c1_1163
60.7 c1_1124
61.1 c1_1077
61.5 c1_1218 c1_1250

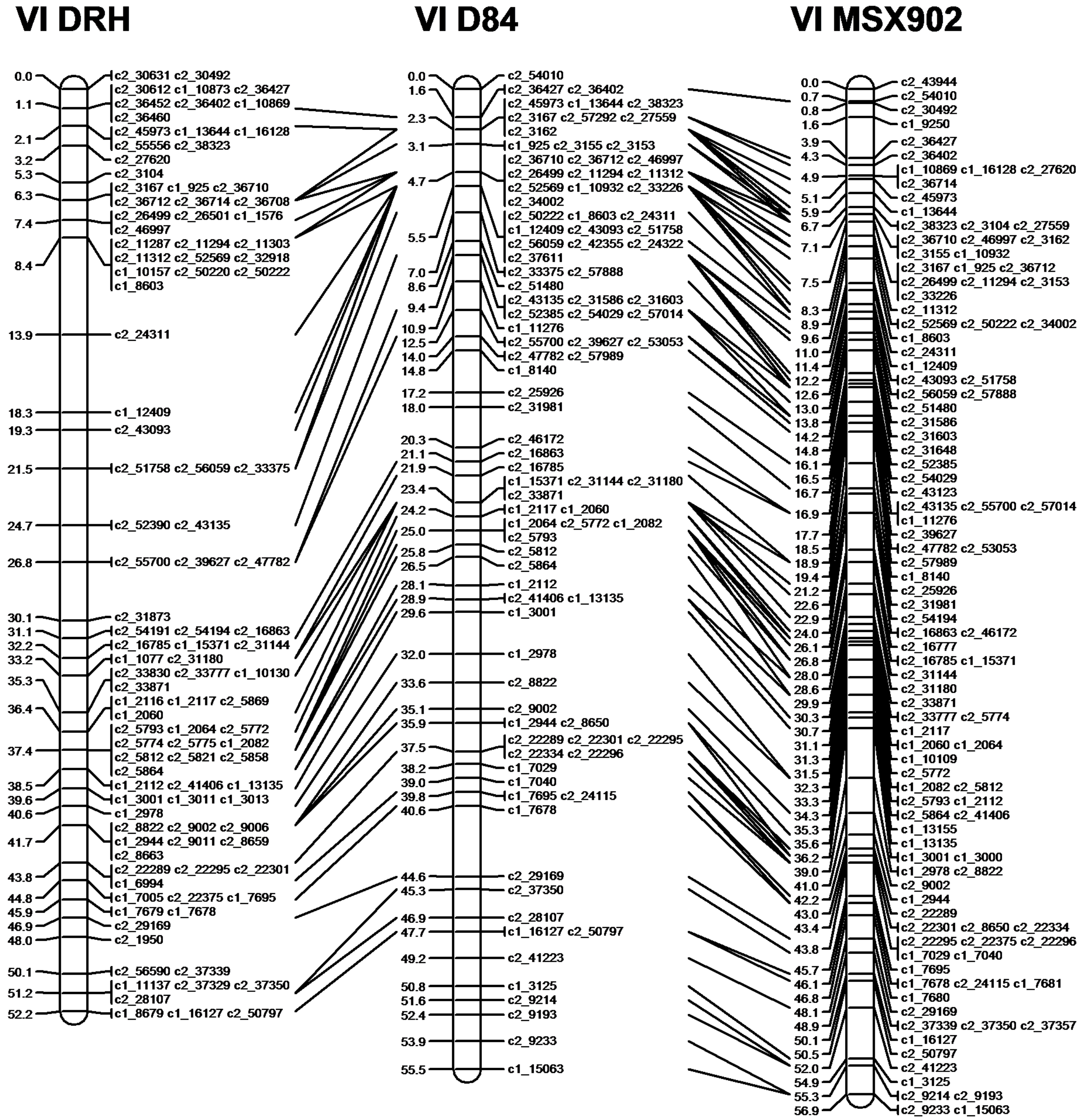
VI DRH
0.0
1.1
2.1
3.2
5.3
6.3
7.4
8.4
13.9
18.3
19.3
21.5
24.7
26.8
30.1
31.1
32.2
33.2
35.3
36.4
37.4
38.5
39.6
40.6
41.7
43.8
44.8
45.9
46.9
48.0
50.1
51.2
52.2
c2_30631 c2_30492
c2_30612 c1_10873 c2_36427
c2_36452 c2_36402 c1_10869
c2_36460
c2_45973 c1_13644 c1_16128
c2_55556 c2_38323
c2_27620
c2_3104
c2_3167 c1_925 c2_36710
c2_36712 c2_36714 c2_36708
c2_26499 c2_26501 c1_1576
c2_46997
c2_11287 c2_11294 c2_11303
c2_11312 c2_52569 c2_32918
c1_10157 c2_50220 c2_50222
c1_8603
c2_24311
c1_12409
c2_43093
c2_51758 c2_56059 c2_33375
c2_52390 c2_43135
c2_55700 c2_39627 c2_47782
c2_31873
c2_54191 c2_54194 c2_16863
c2_16785 c1_15371 c2_31144
c1_1077 c2_31180
c2_33830 c2_33777 c1_10130
c2_33871
c1_2116 c1_2117 c2_5869
c1_2060
c2_5793 c1_2064 c2_5772
c2_5774 c2_5775 c1_2082
c2_5812 c2_5821 c2_5858
c2_5864
c1_2112 c2_41406 c1_13135
c1_3001 c1_3011 c1_3013
c1_2978
c2_8822 c2_9002 c2_9006
c1_2944 c2_9011 c2_8659
c2_8663
c2_22289 c2_22295 c2_22301
c1_6994
c1_7005 c2_22375 c1_7695
c1_7679 c1_7678
c2_29169
c2_1950
c2_56590 c2_37339
c1_11137 c2_37329 c2_37350
c2_28107
c1_8679 c1_16127 c2_50797
VI D84
0.0
1.6
2.3
3.1
4.7
5.5
7.0
8.6
9.4
10.9
12.5
14.0
14.8
17.2
18.0
20.3
21.1
21.9
23.4
24.2
25.0
25.8
26.5
28.1
28.9
29.6
32.0
33.6
35.1
35.9
37.5
38.2
39.0
39.8
40.6
44.6
45.3
46.9
47.7
49.2
50.8
51.6
52.4
53.9
55.5
c2_54010
c2_36427 c2_36402
c2_45973 c1_13644 c2_38323
c2_3167 c2_57292 c2_27559
c2_3162
c1_925 c2_3155 c2_3153
c2_36710 c2_36712 c2_46997
c2_26499 c2_11294 c2_11312
c2_52569 c1_10932 c2_33226
c2_34002
c2_50222 c1_8603 c2_24311
c1_12409 c2_43093 c2_51758
c2_56059 c2_42355 c2_24322
c2_37611
c2_33375 c2_57888
c2_51480
c2_43135 c2_31586 c2_31603
c2_52385 c2_54029 c2_57014
c1_11276
c2_55700 c2_39627 c2_53053
c2_47782 c2_57989
c1_8140
c2_25926
c2_31981
c2_46172
c2_16863
c2_16785
c1_15371 c2_31144 c2_31180
c2_33871
c1_2117 c1_2060
c1_2064 c2_5772 c1_2082
c2_5793
c2_5812
c2_5864
c1_2112
c2_41406 c1_13135
c1_3001
c1_2978
c2_8822
c2_9002
c1_2944 c2_8650
c2_22289 c2_22301 c2_22295
c2_22334 c2_22296
c1_7029
c1_7040
c1_7695 c2_24115
c1_7678
c2_29169
c2_37350
c2_28107
c1_16127 c2_50797
c2_41223
c1_3125
c2_9214
c2_9193
c2_9233
c1_15063
VI MSX902
0.0
0.7
0.8
1.6
3.9
4.3
4.9
5.1
5.9
6.7
7.1
7.5
8.3
8.9
9.6
11.0
11.4
12.2
12.6
13.0
13.8
14.2
14.8
16.1
16.5
16.7
16.9
17.7
18.5
18.9
19.4
21.2
22.6
22.9
24.0
26.1
26.8
28.0
28.6
29.9
30.3
30.7
31.1
31.3
31.5
32.3
33.3
34.3
35.3
35.6
36.2
39.0
41.0
42.2
43.0
43.4
43.8
45.7
46.1
46.8
48.1
48.9
50.1
50.5
52.0
54.9
55.3
56.9
c2_43944
c2_54010
c2_30492
c1_9250
c2_36427
c2_36402
c1_10869 c1_16128 c2_27620
c2_36714
c2_45973
c1_13644
c2_38323 c2_3104 c2_27559
c2_36710 c2_46997 c2_3162
c2_3155 c1_10932
c2_3167 c1_925 c2_36712
c2_26499 c2_11294 c2_3153
c2_33226
c2_11312
c2_52569 c2_50222 c2_34002
c1_8603
c2_24311
c1_12409
c2_43093 c2_51758
c2_56059 c2_57888
c2_51480
c2_31586
c2_31603
c2_31648
c2_52385
c2_54029
c2_43123
c2_43135 c2_55700 c2_57014
c1_11276
c2_39627
c2_47782 c2_53053
c2_57989
c1_8140
c2_25926
c2_31981
c2_54194
c2_16863 c2_46172
c2_16777
c2_16785 c1_15371
c2_31144
c2_31180
c2_33871
c2_33777 c2_5774
c1_2117
c1_2060 c1_2064
c1_10109
c2_5772
c1_2082 c2_5812
c2_5793 c1_2112
c2_5864 c2_41406
c1_13155
c1_13135
c1_3001 c1_3000
c1_2978 c2_8822
c2_9002
c1_2944
c2_22289
c2_22301 c2_8650 c2_22334
c2_22295 c2_22375 c2_22296
c1_7029 c1_7040
c1_7695
c1_7678 c2_24115 c1_7681
c1_7680
c2_29169
c2_37339 c2_37350 c2_37357
c1_16127
c2_50797
c2_41223
c1_3125
c2_9214 c2_9193
c2_9233 c1_15063

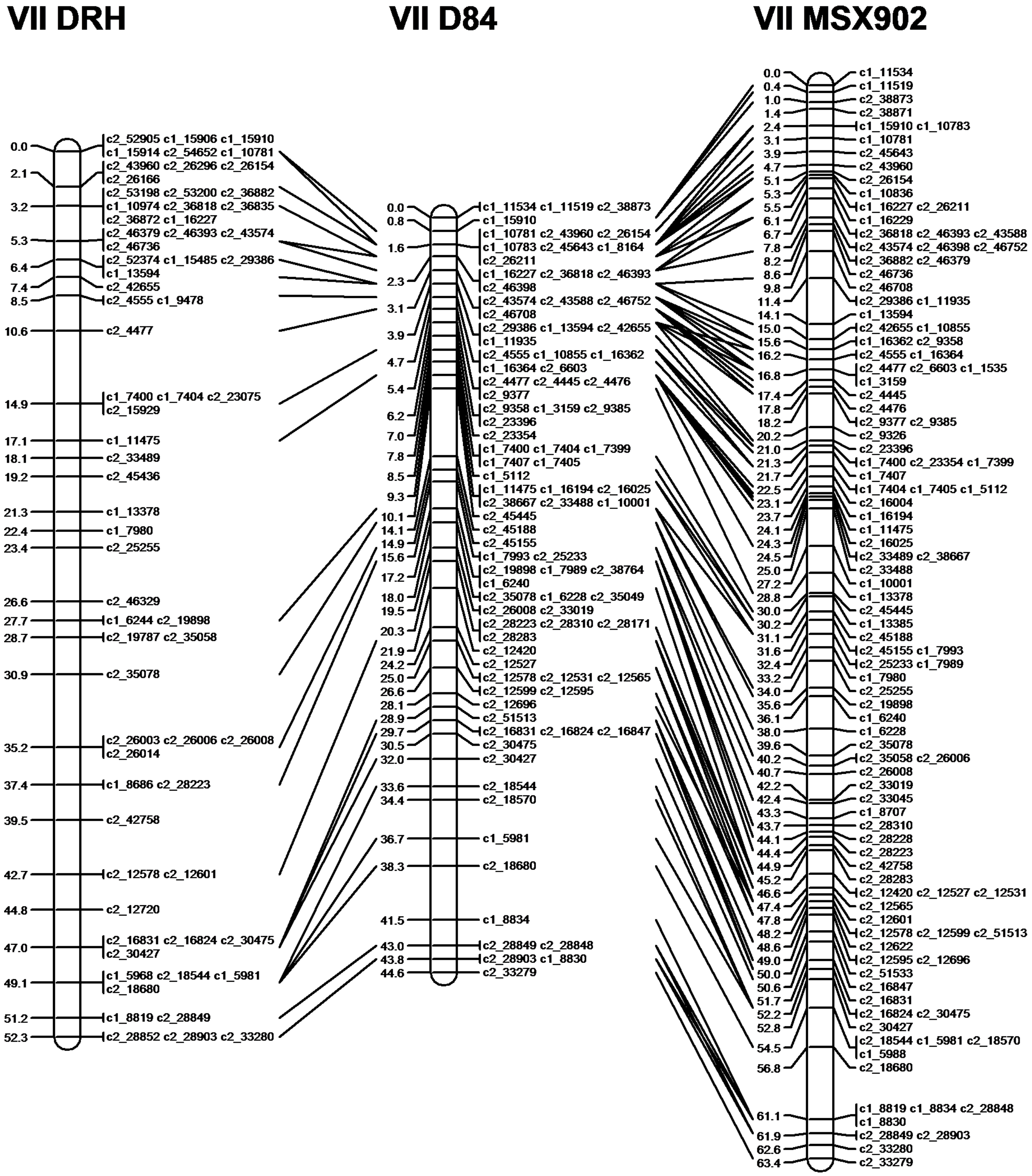

VII DRH
0.0 c2_52905 c1_15906 c1_15910
c1_15914 c2_54652 c1_10781
2.1 c2_43960 c2_26296 c2_26154
c2_26166
3.2 c2_53198 c2_53200 c2_36882
c1_10974 c2_36818 c2_36835
c2_36872 c1_16227
5.3 c2_46379 c2_46393 c2_43574
c2_46736
6.4 c2_52374 c1_15485 c2_29386
c1_13594
7.4 c2_42655
8.5 c2_4555 c1_9478
10.6 c2_4477
14.9 c1_7400 c1_7404 c2_23075
c2_15929
17.1 c1_11475
18.1 c2_33489
19.2 c2_45436
21.3 c1_13378
22.4 c1_7980
23.4 c2_25255
26.6 c2_46329
27.7 c1_6244 c2_19898
28.7 c2_19787 c2_35058
30.9 c2_35078
35.2 c2_26003 c2_26006 c2_26008
c2_26014
37.4 c1_8686 c2_28223
39.5 c2_42758
42.7 c2_12578 c2_12601
44.8 c2_12720
47.0 c2_16831 c2_16824 c2_30475
c2_30427
49.1 c1_5968 c2_18544 c1_5981
c2_18680
51.2 c1_8819 c2_28849
52.3 c2_28852 c2_28903 c2_33280
VII D84
0.0
0.8
1.6
2.3
3.1
3.9
4.7
5.4
6.2
7.0
7.8
8.5
9.3
10.1
14.1
14.9
15.6
17.2
18.0
19.5
20.3
21.9
24.2
25.0
26.6
28.1
28.9
29.7
30.5
32.0
33.6
34.4
36.7
38.3
41.5
43.0
43.8
44.6
c1_11534 c1_11519 c2_38873
c1_15910
c1_10781 c2_43960 c2_26154
c1_10783 c2_45643 c1_8164
c2_26211
c1_16227 c2_36818 c2_46393
c2_46398
c2_43574 c2_43588 c2_46752
c2_46708
c2_29386 c1_13594 c2_42655
c1_11935
c2_4555 c1_10855 c1_16362
c1_16364 c2_6603
c2_4477 c2_4445 c2_4476
c2_9377
c2_9358 c1_3159 c2_9385
c2_23396
c2_23354
c1_7400 c1_7404 c1_7399
c1_7407 c1_7405
c1_5112
c1_11475 c1_16194 c2_16025
c2_38667 c2_33488 c1_10001
c2_45445
c2_45188
c2_45155
c1_7993 c2_25233
c2_19898 c1_7989 c2_38764
c1_6240
c2_35078 c1_6228 c2_35049
c2_26008 c2_33019
c2_28223 c2_28310 c2_28171
c2_28283
c2_12420
c2_12527
c2_12578 c2_12531 c2_12565
c2_12599 c2_12595
c2_12696
c2_51513
c2_16831 c2_16824 c2_16847
c2_30475
c2_30427
c2_18544
c2_18570
c1_5981
c2_18680
c1_8834
c2_28849 c2_28848
c2_28903 c1_8830
c2_33279
VII MSX902
0.0
0.4
1.0
1.4
2.4
3.1
3.9
4.7
5.1
5.3
5.5
6.1
6.7
7.8
8.2
8.6
9.8
11.4
14.1
15.0
15.6
16.2
16.8
17.4
17.8
18.2
20.2
21.0
21.3
21.7
22.5
23.1
23.7
24.1
24.3
24.5
25.0
27.2
28.8
30.0
30.2
31.1
31.6
32.4
33.2
34.0
35.6
36.1
38.0
39.6
40.2
40.7
42.2
42.4
43.3
43.7
44.1
44.4
44.9
45.2
46.6
47.4
47.8
48.2
48.6
49.0
50.0
50.6
51.7
52.2
52.8
54.5
56.8
61.1
61.9
62.6
63.4
c1_11534
c1_11519
c2_38873
c2_38871
c1_15910 c1_10783
c1_10781
c2_45643
c2_43960
c2_26154
c1_10836
c1_16227 c2_26211
c1_16229
c2_36818 c2_46393 c2_43588
c2_43574 c2_46398 c2_46752
c2_36882 c2_46379
c2_46736
c2_46708
c2_29386 c1_11935
c1_13594
c2_42655 c1_10855
c1_16362 c2_9358
c2_4555 c1_16364
c2_4477 c2_6603 c1_1535
c1_3159
c2_4445
c2_4476
c2_9377 c2_9385
c2_9326
c2_23396
c1_7400 c2_23354 c1_7399
c1_7407
c1_7404 c1_7405 c1_5112
c2_16004
c1_16194
c1_11475
c2_16025
c2_33489 c2_38667
c2_33488
c1_10001
c1_13378
c2_45445
c1_13385
c2_45188
c2_45155 c1_7993
c2_25233 c1_7989
c1_7980
c2_25255
c2_19898
c1_6240
c1_6228
c2_35078
c2_35058 c2_26006
c2_26008
c2_33019
c2_33045
c1_8707
c2_28310
c2_28228
c2_28223
c2_42758
c2_28283
c2_12420 c2_12527 c2_12531
c2_12565
c2_12601
c2_12578 c2_12599 c2_51513
c2_12622
c2_12595 c2_12696
c2_51533
c2_16847
c2_16831
c2_16824 c2_30475
c2_30427
c2_18544 c1_5981 c2_18570
c1_5988
c2_18680
c1_8819 c1_8834 c2_28848
c1_8830
c2_28849 c2_28903
c2_33280
c2_33279

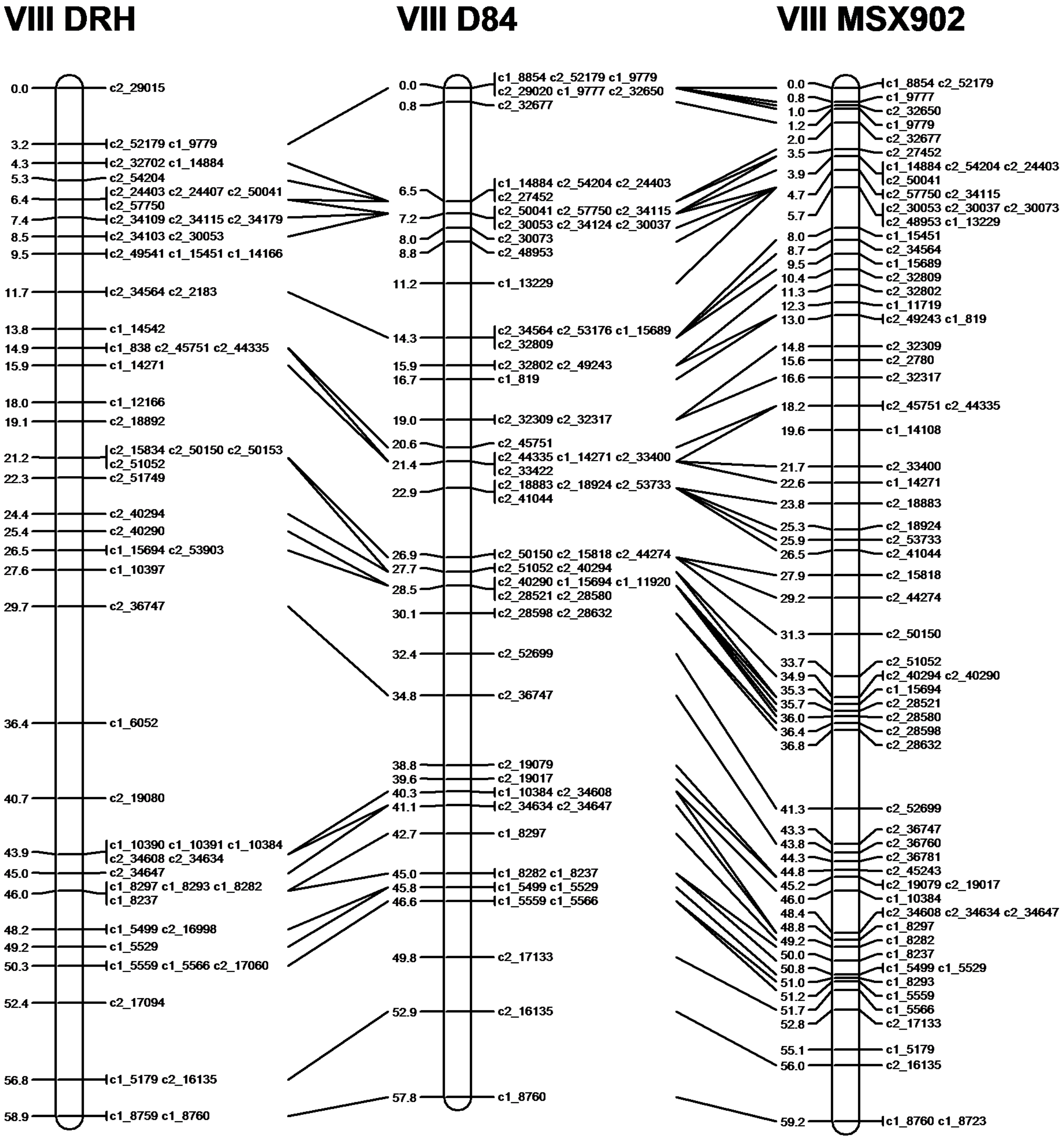

VIII DRH
0.0 c2_29015
3.2 c2_52179 c1_9779
4.3 c2_32702 c1_14884
5.3 c2_54204
6.4 c2_24403 c2_24407 c2_50041 c2_57750
7.4 c2_34109 c2_34115 c2_34179
8.5 c2_34103 c2_30053
9.5 c2_49541 c1_15451 c1_14166
11.7 c2_34564 c2_2183
13.8 c1_14542
14.9 c1_838 c2_45751 c2_44335
15.9 c1_14271
18.0 c1_12166
19.1 c2_18892
21.2 c2_15834 c2_50150 c2_50153 c2_51052
22.3 c2_51749
24.4 c2_40294
25.4 c2_40290
26.5 c1_15694 c2_53903
27.6 c1_10397
29.7 c2_36747
36.4 c1_6052
40.7 c2_19080
43.9 c1_10390 c1_10391 c1_10384 c2_34608 c2_34634
45.0 c2_34647
46.0 c1_8297 c1_8293 c1_8282 c1_8237
48.2 c1_5499 c2_16998
49.2 c1_5529
50.3 c1_5559 c1_5566 c2_17060
52.4 c2_17094
56.8 c1_5179 c2_16135
58.9 c1_8759 c1_8760
VIII D84
0.0 c1_8854 c2_52179 c1_9779 c2_29020 c1_9777 c2_32650
0.8 c2_32677
6.5 c1_14884 c2_54204 c2_24403 c2_27452
7.2 c2_50041 c2_57750 c2_34115 c2_30053 c2_34124 c2_30037
8.0 c2_30073
8.8 c2_48953
11.2 c1_13229
14.3 c2_34564 c2_53176 c1_15689 c2_32809
15.9 c2_32802 c2_49243
16.7 c1_819
19.0 c2_32309 c2_32317
20.6 c2_45751
21.4 c2_44335 c1_14271 c2_33400 c2_33422
22.9 c2_18883 c2_18924 c2_53733 c2_41044
26.9 c2_50150 c2_15818 c2_44274
27.7 c2_51052 c2_40294
28.5 c2_40290 c1_15694 c1_11920 c2_28521 c2_28580
30.1 c2_28598 c2_28632
32.4 c2_52699
34.8 c2_36747
38.8 c2_19079
39.6 c2_19017
40.3 c1_10384 c2_34608
41.1 c2_34634 c2_34647
42.7 c1_8297
45.0 c1_8282 c1_8237
45.8 c1_5499 c1_5529
46.6 c1_5559 c1_5566
49.8 c2_17133
52.9 c2_16135
57.8 c1_8760
VIII MSX902
0.0 c1_8854 c2_52179
0.8 c1_9777
1.0 c2_32650
1.2 c1_9779
2.0 c2_32677
3.5 c2_27452
3.9 c1_14884 c2_54204 c2_24403 c2_50041
4.7 c2_57750 c2_34115
5.7 c2_30053 c2_30037 c2_30073 c2_48953 c1_13229
8.0 c1_15451
8.7 c2_34564
9.5 c1_15689
10.4 c2_32809
11.3 c2_32802
12.3 c1_11719
13.0 c2_49243 c1_819
14.8 c2_32309
15.6 c2_2780
16.6 c2_32317
18.2 c2_45751 c2_44335
19.6 c1_14108
21.7 c2_33400
22.6 c1_14271
23.8 c2_18883
25.3 c2_18924
25.9 c2_53733
26.5 c2_41044
27.9 c2_15818
29.2 c2_44274
31.3 c2_50150
33.7 c2_51052
34.9 c2_40294 c2_40290
35.3 c1_15694
35.7 c2_28521
36.0 c2_28580
36.4 c2_28598
36.8 c2_28632
41.3 c2_52699
43.3 c2_36747
43.8 c2_36760
44.3 c2_36781
44.8 c2_45243
45.2 c2_19079 c2_19017
46.0 c1_10384
48.4 c2_34608 c2_34634 c2_34647
48.8 c1_8297
49.2 c1_8282
50.0 c1_8237
50.8 c1_5499 c1_5529
51.0 c1_8293
51.2 c1_5559
51.7 c1_5566
52.8 c2_17133
55.1 c1_5179
56.0 c2_16135
59.2 c1_8760 c1_8723

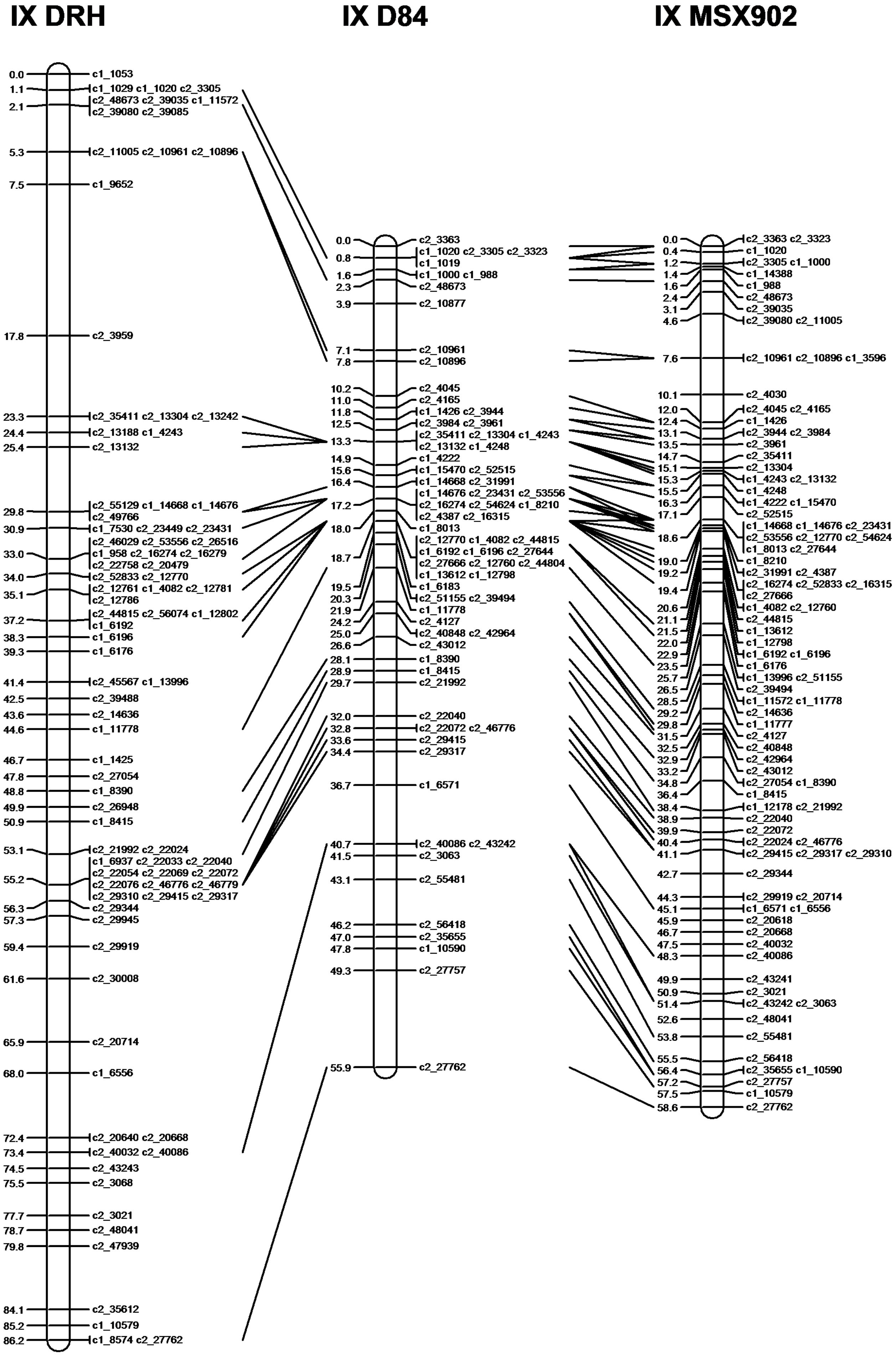

IX DRH
0.0 c1_1053
1.1 c1_1029 c1_1020 c2_3305
2.1 c2_48673 c2_39035 c1_11572
c2_39080 c2_39085
5.3 c2_11005 c2_10961 c2_10896
7.5 c1_9652
17.8 c2_3959
23.3 c2_35411 c2_13304 c2_13242
24.4 c2_13188 c1_4243
25.4 c2_13132
29.8 c2_55129 c1_14668 c1_14676
c2_49766
30.9 c1_7530 c2_23449 c2_23431
c2_46029 c2_53556 c2_26516
33.0 c1_958 c2_16274 c2_16279
c2_22758 c2_20479
34.0 c2_52833 c2_12770
35.1 c2_12761 c1_4082 c2_12781
c2_12786
37.2 c2_44815 c2_56074 c1_12802
c1_6192
38.3 c1_6196
39.3 c1_6176
41.4 c2_45567 c1_13996
42.5 c2_39488
43.6 c2_14636
44.6 c1_11778
46.7 c1_1425
47.8 c2_27054
48.8 c1_8390
49.9 c2_26948
50.9 c1_8415
53.1 c2_21992 c2_22024
c1_6937 c2_22033 c2_22040
55.2 c2_22054 c2_22069 c2_22072
c2_22076 c2_46776 c2_46779
c2_29310 c2_29415 c2_29317
56.3 c2_29344
57.3 c2_29945
59.4 c2_29919
61.6 c2_30008
65.9 c2_20714
68.0 c1_6556
72.4 c2_20640 c2_20668
73.4 c2_40032 c2_40086
74.5 c2_43243
75.5 c2_3068
77.7 c2_3021
78.7 c2_48041
79.8 c2_47939
84.1 c2_35612
85.2 c1_10579
86.2 c1_8574 c2_27762
IX D84
0.0 c2_3363
0.8 c1_1020 c2_3305 c2_3323
c1_1019
1.6 c1_1000 c1_988
2.3 c2_48673
3.9 c2_10877
7.1 c2_10961
7.8 c2_10896
10.2 c2_4045
11.0 c2_4165
11.8 c1_1426 c2_3944
12.5 c2_3984 c2_3961
13.3 c2_35411 c2_13304 c1_4243
c2_13132 c1_4248
14.9 c1_4222
15.6 c1_15470 c2_52515
16.4 c1_14668 c2_31991
c1_14676 c2_23431 c2_53556
17.2 c2_16274 c2_54624 c1_8210
c2_4387 c2_16315
18.0 c1_8013
c2_12770 c1_4082 c2_44815
18.7 c1_6192 c1_6196 c2_27644
c2_27666 c2_12760 c2_44804
c1_13612 c1_12798
19.5 c1_6183
20.3 c2_51155 c2_39494
21.9 c1_11778
24.2 c2_4127
25.0 c2_40848 c2_42964
26.6 c2_43012
28.1 c1_8390
28.9 c1_8415
29.7 c2_21992
32.0 c2_22040
32.8 c2_22072 c2_46776
33.6 c2_29415
34.4 c2_29317
36.7 c1_6571
40.7 c2_40086 c2_43242
41.5 c2_3063
43.1 c2_55481
46.2 c2_56418
47.0 c2_35655
47.8 c1_10590
49.3 c2_27757
55.9 c2_27762
IX MSX902
0.0 c2_3363 c2_3323
0.4 c1_1020
1.2 c2_3305 c1_1000
1.4 c1_14388
1.6 c1_988
2.4 c2_48673
3.1 c2_39035
4.6 c2_39080 c2_11005
7.6 c2_10961 c2_10896 c1_3596
10.1 c2_4030
12.0 c2_4045 c2_4165
12.4 c1_1426
13.1 c2_3944 c2_3984
13.5 c2_3961
14.7 c2_35411
15.1 c2_13304
15.3 c1_4243 c2_13132
15.5 c1_4248
16.3 c1_4222 c1_15470
17.1 c2_52515
18.6 c1_14668 c1_14676 c2_23431
c2_53556 c2_12770 c2_54624
c1_8013 c2_27644
19.0 c1_8210
19.2 c2_31991 c2_4387
19.4 c2_16274 c2_52833 c2_16315
c2_27666
20.6 c1_4082 c2_12760
21.1 c2_44815
21.5 c1_13612
22.0 c1_12798
22.9 c1_6192 c1_6196
23.5 c1_6176
25.7 c1_13996 c2_51155
26.5 c2_39494
28.5 c1_11572 c1_11778
29.2 c2_14636
29.8 c1_11777
31.5 c2_4127
32.5 c2_40848
32.9 c2_42964
33.2 c2_43012
34.8 c2_27054 c1_8390
36.4 c1_8415
38.4 c1_12178 c2_21992
38.9 c2_22040
39.9 c2_22072
40.4 c2_22024 c2_46776
41.1 c2_29415 c2_29317 c2_29310
42.7 c2_29344
44.3 c2_29919 c2_20714
45.1 c1_6571 c1_6556
45.9 c2_20618
46.7 c2_20668
47.5 c2_40032
48.3 c2_40086
49.9 c2_43241
50.9 c2_3021
51.4 c2_43242 c2_3063
52.6 c2_48041
53.8 c2_55481
55.5 c2_56418
56.4 c2_35655 c1_10590
57.2 c2_27757
57.5 c1_10579
58.6 c2_27762

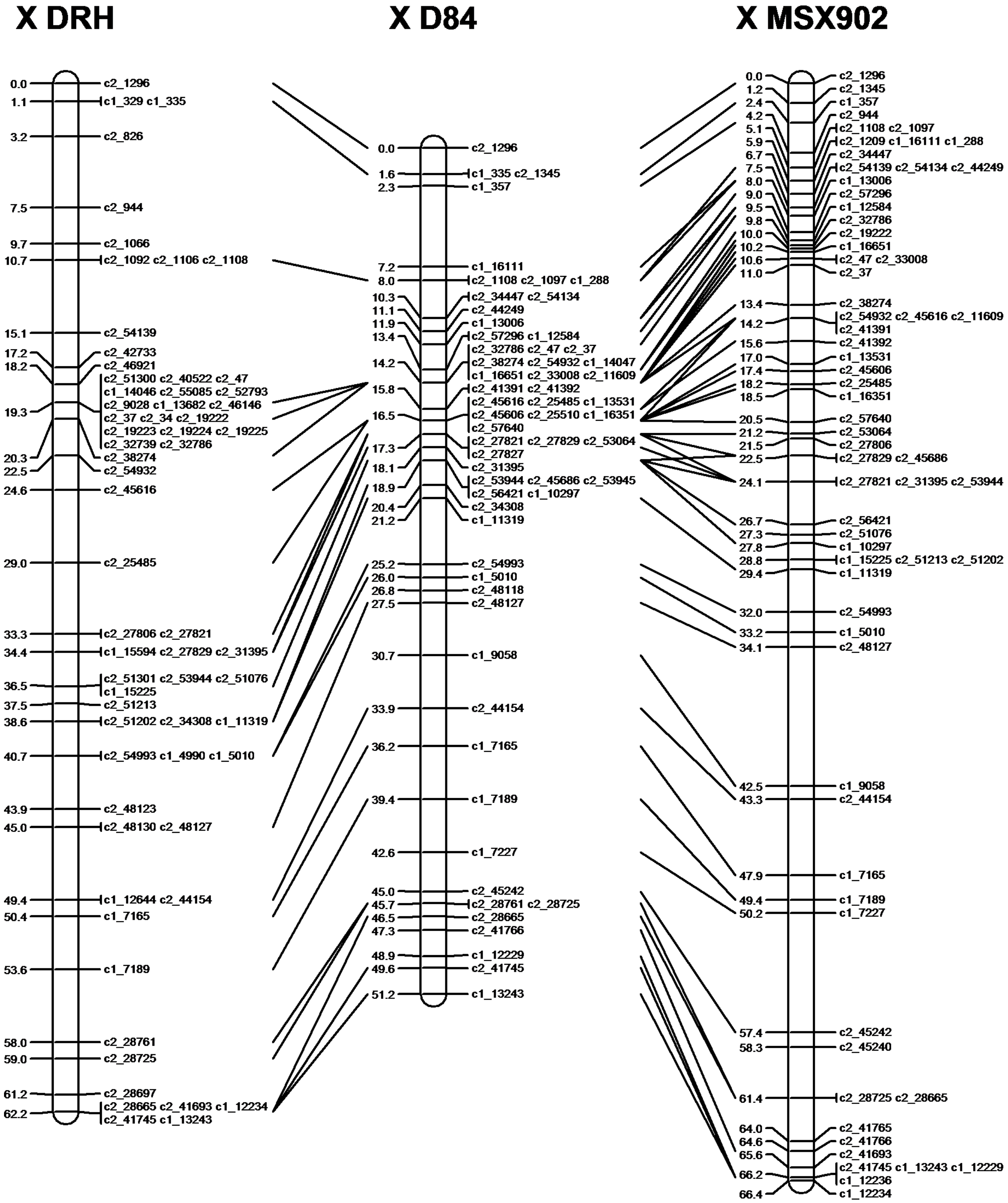

X DRH
X D84
X MSX902
0.0 c2_1296
1.1 c1_329 c1_335
3.2 c2_826
7.5 c2_944
9.7 c2_1066
10.7 c2_1092 c2_1106 c2_1108
15.1 c2_54139
17.2 c2_42733
18.2 c2_46921
19.3 c2_51300 c2_40522 c2_47 c1_14046 c2_55085 c2_52793 c2_9028 c1_13682 c2_46146 c2_37 c2_34 c2_19222 c2_19223 c2_19224 c2_19225 c2_32739 c2_32786
20.3 c2_38274
22.5 c2_54932
24.6 c2_45616
29.0 c2_25485
33.3 c2_27806 c2_27821
34.4 c1_15594 c2_27829 c2_31395
36.5 c2_51301 c2_53944 c2_51076 c1_15225
37.5 c2_51213
38.6 c2_51202 c2_34308 c1_11319
40.7 c2_54993 c1_4990 c1_5010
43.9 c2_48123
45.0 c2_48130 c2_48127
49.4 c1_12644 c2_44154
50.4 c1_7165
53.6 c1_7189
58.0 c2_28761
59.0 c2_28725
61.2 c2_28697
62.2 c2_28665 c2_41693 c1_12234 c2_41745 c1_13243
0.0 c2_1296
1.6 c1_335 c2_1345
2.3 c1_357
7.2 c1_16111
8.0 c2_1108 c2_1097 c1_288
10.3 c2_34447 c2_54134
11.1 c2_44249
11.9 c1_13006
13.4 c2_57296 c1_12584
14.2 c2_32786 c2_47 c2_37 c2_38274 c2_54932 c1_14047 c1_16651 c2_33008 c2_11609
15.8 c2_41391 c2_41392
16.5 c2_45616 c2_25485 c1_13531 c2_45606 c2_25510 c1_16351 c2_57640
17.3 c2_27821 c2_27829 c2_53064 c2_27827
18.1 c2_31395
18.9 c2_53944 c2_45686 c2_53945 c2_56421 c1_10297
20.4 c2_34308
21.2 c1_11319
25.2 c2_54993
26.0 c1_5010
26.8 c2_48118
27.5 c2_48127
30.7 c1_9058
33.9 c2_44154
36.2 c1_7165
39.4 c1_7189
42.6 c1_7227
45.0 c2_45242
45.7 c2_28761 c2_28725
46.5 c2_28665
47.3 c2_41766
48.9 c1_12229
49.6 c2_41745
51.2 c1_13243
0.0 c2_1296
1.2 c2_1345
2.4 c1_357
4.2 c2_944
5.1 c2_1108 c2_1097
5.9 c2_1209 c1_16111 c1_288
6.7 c2_34447
7.5 c2_54139 c2_54134 c2_44249
8.0 c1_13006
9.0 c2_57296
9.5 c1_12584
9.8 c2_32786
10.0 c2_19222
10.2 c1_16651
10.6 c2_47 c2_33008
11.0 c2_37
13.4 c2_38274
14.2 c2_54932 c2_45616 c2_11609 c2_41391
15.6 c2_41392
17.0 c1_13531
17.4 c2_45606
18.2 c2_25485
18.5 c1_16351
20.5 c2_57640
21.2 c2_53064
21.5 c2_27806
22.5 c2_27829 c2_45686
24.1 c2_27821 c2_31395 c2_53944
26.7 c2_56421
27.3 c2_51076
27.8 c1_10297
28.8 c1_15225 c2_51213 c2_51202
29.4 c1_11319
32.0 c2_54993
33.2 c1_5010
34.1 c2_48127
42.5 c1_9058
43.3 c2_44154
47.9 c1_7165
49.4 c1_7189
50.2 c1_7227
57.4 c2_45242
58.3 c2_45240
61.4 c2_28725 c2_28665
64.0 c2_41765
64.6 c2_41766
65.6 c2_41693
66.2 c2_41745 c1_13243 c1_12229 c1_12236
66.4 c1_12234

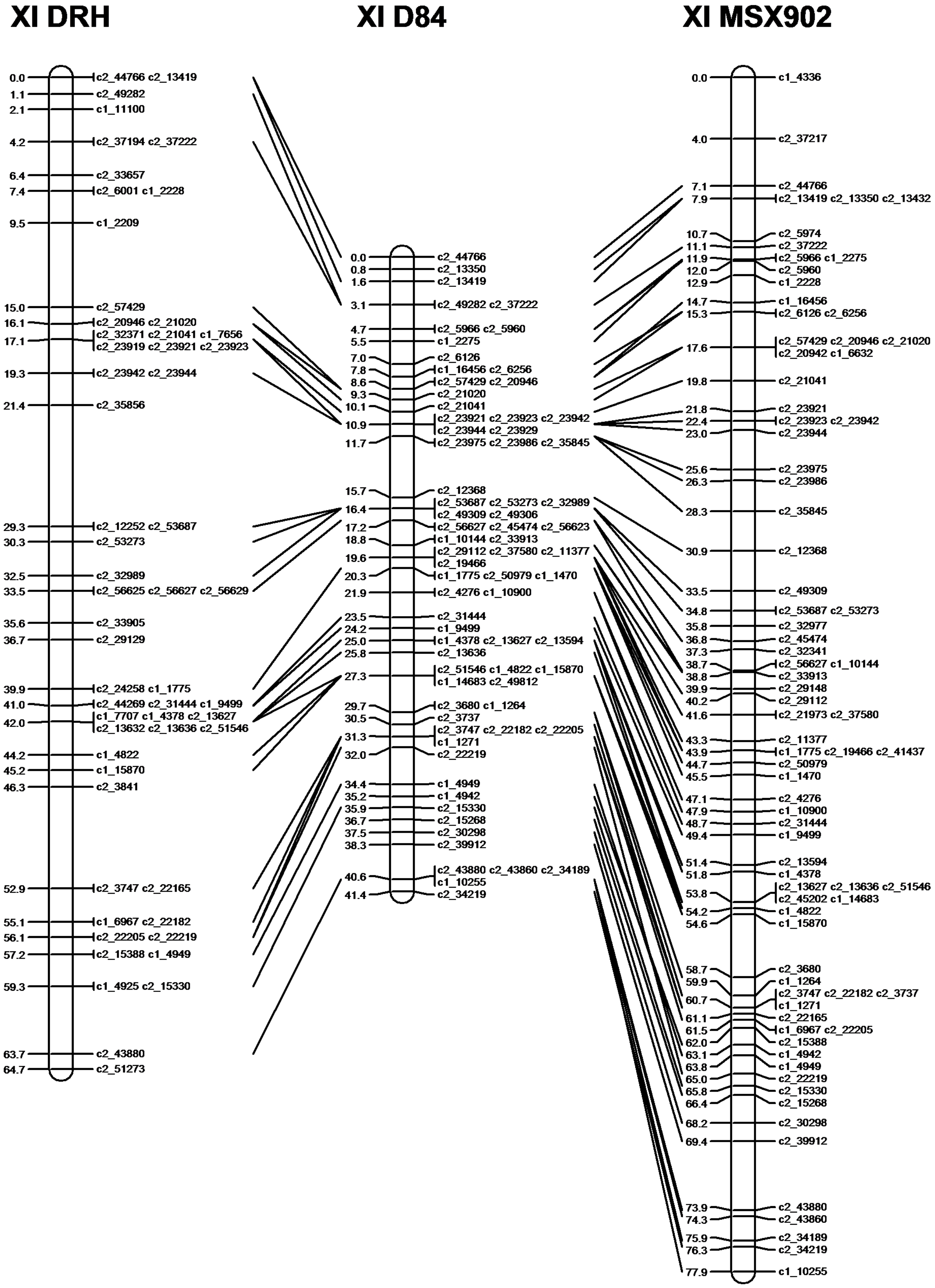
XI DRH
0.0 c2_44766 c2_13419
1.1 c2_49282
2.1 c1_11100
4.2 c2_37194 c2_37222
6.4 c2_33657
7.4 c2_6001 c1_2228
9.5 c1_2209
15.0 c2_57429
16.1 c2_20946 c2_21020
17.1 c2_32371 c2_21041 c1_7656 c2_23919 c2_23921 c2_23923
19.3 c2_23942 c2_23944
21.4 c2_35856
29.3 c2_12252 c2_53687
30.3 c2_53273
32.5 c2_32989
33.5 c2_56625 c2_56627 c2_56629
35.6 c2_33905
36.7 c2_29129
39.9 c2_24258 c1_1775
41.0 c2_44269 c2_31444 c1_9499
42.0 c1_7707 c1_4378 c2_13627 c2_13632 c2_13636 c2_51546
44.2 c1_4822
45.2 c1_15870
46.3 c2_3841
52.9 c2_3747 c2_22165
55.1 c1_6967 c2_22182
56.1 c2_22205 c2_22219
57.2 c2_15388 c1_4949
59.3 c1_4925 c2_15330
63.7 c2_43880
64.7 c2_51273
XI D84
0.0 c2_44766
0.8 c2_13350
1.6 c2_13419
3.1 c2_49282 c2_37222
4.7 c2_5966 c2_5960
5.5 c1_2275
7.0 c2_6126
7.8 c1_16456 c2_6256
8.6 c2_57429 c2_20946
9.3 c2_21020
10.1 c2_21041
10.9 c2_23921 c2_23923 c2_23942 c2_23944 c2_23929
11.7 c2_23975 c2_23986 c2_35845
15.7 c2_12368
16.4 c2_53687 c2_53273 c2_32989 c2_49309 c2_49306
17.2 c2_56627 c2_45474 c2_56623
18.8 c1_10144 c2_33913
19.6 c2_29112 c2_37580 c2_11377 c2_19466
20.3 c1_1775 c2_50979 c1_1470
21.9 c2_4276 c1_10900
23.5 c2_31444
24.2 c1_9499
25.0 c1_4378 c2_13627 c2_13594
25.8 c2_13636
27.3 c2_51546 c1_4822 c1_15870 c1_14683 c2_49812
29.7 c2_3680 c1_1264
30.5 c2_3737
31.3 c2_3747 c2_22182 c2_22205 c1_1271
32.0 c2_22219
34.4 c1_4949
35.2 c1_4942
35.9 c2_15330
36.7 c2_15268
37.5 c2_30298
38.3 c2_39912
40.6 c2_43880 c2_43860 c2_34189 c1_10255
41.4 c2_34219
XI MSX902
0.0 c1_4336
4.0 c2_37217
7.1 c2_44766
7.9 c2_13419 c2_13350 c2_13432
10.7 c2_5974
11.1 c2_37222
11.9 c2_5966 c1_2275
12.0 c2_5960
12.9 c1_2228
14.7 c1_16456
15.3 c2_6126 c2_6256
17.6 c2_57429 c2_20946 c2_21020 c2_20942 c1_6632
19.8 c2_21041
21.8 c2_23921
22.4 c2_23923 c2_23942
23.0 c2_23944
25.6 c2_23975
26.3 c2_23986
28.3 c2_35845
30.9 c2_12368
33.5 c2_49309
34.8 c2_53687 c2_53273
35.8 c2_32977
36.8 c2_45474
37.3 c2_32341
38.7 c2_56627 c1_10144
38.8 c2_33913
39.9 c2_29148
40.2 c2_29112
41.6 c2_21973 c2_37580
43.3 c2_11377
43.9 c1_1775 c2_19466 c2_41437
44.7 c2_50979
45.5 c1_1470
47.1 c2_4276
47.9 c1_10900
48.7 c2_31444
49.4 c1_9499
51.4 c2_13594
51.8 c1_4378
53.8 c2_13627 c2_13636 c2_51546 c2_45202 c1_14683
54.2 c1_4822
54.6 c1_15870
58.7 c2_3680
59.9 c1_1264
60.7 c2_3747 c2_22182 c2_3737 c1_1271
61.1 c2_22165
61.5 c1_6967 c2_22205
62.0 c2_15388
63.1 c1_4942
63.8 c1_4949
65.0 c2_22219
65.8 c2_15330
66.4 c2_15268
68.2 c2_30298
69.4 c2_39912
73.9 c2_43880
74.3 c2_43860
75.9 c2_34189
76.3 c2_34219
77.9 c1_10255

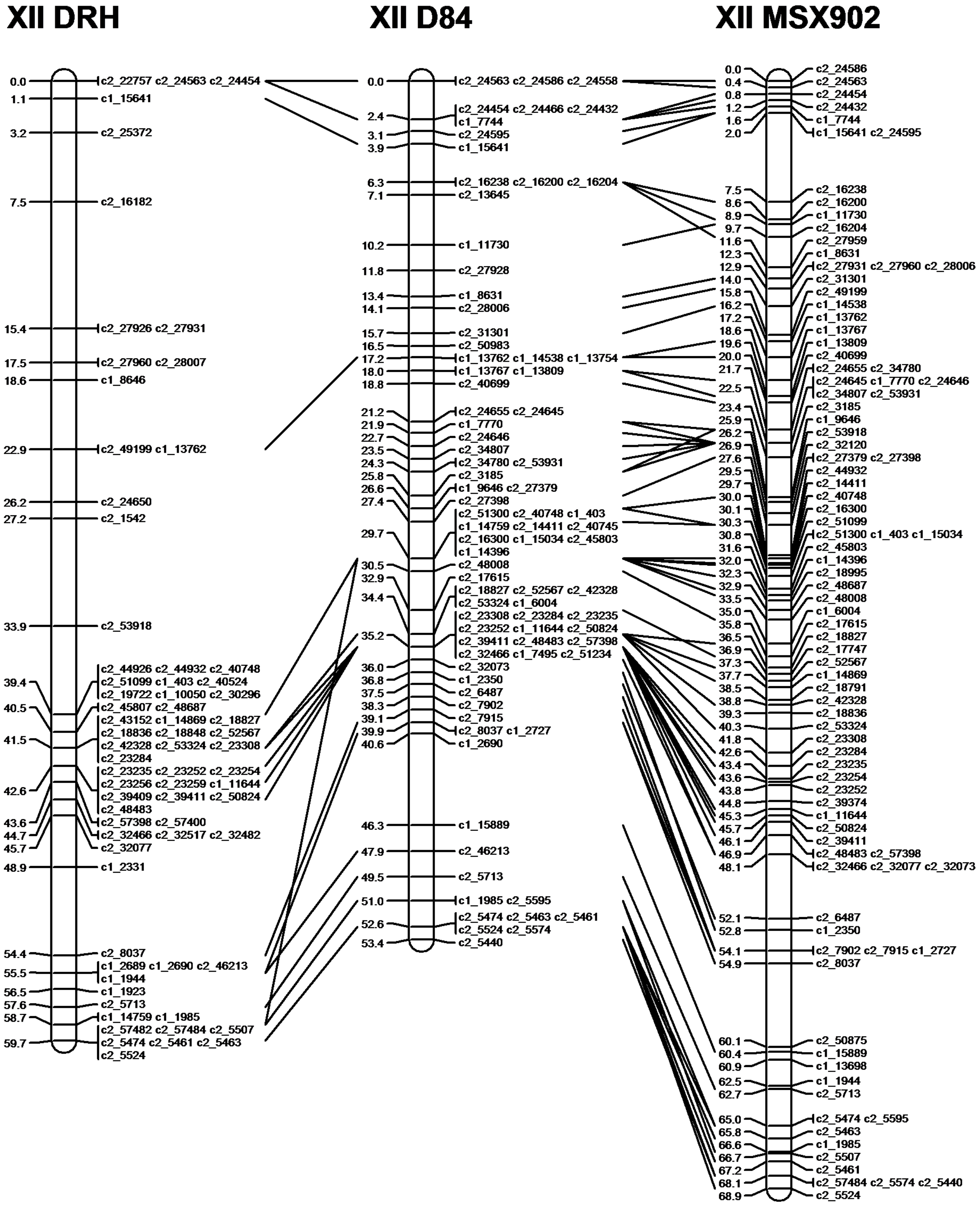
XII DRH
0.0 c2_22757 c2_24563 c2_24454
1.1 c1_15641
3.2 c2_25372
7.5 c2_16182
15.4 c2_27926 c2_27931
17.5 c2_27960 c2_28007
18.6 c1_8646
22.9 c2_49199 c1_13762
26.2 c2_24650
27.2 c2_1542
33.9 c2_53918
39.4 c2_44926 c2_44932 c2_40748 c2_51099 c1_403 c2_40524 c2_19722 c1_10050 c2_30296
40.5 c2_45807 c2_48687
41.5 c2_43152 c1_14869 c2_18827 c2_18836 c2_18848 c2_52567 c2_42328 c2_53324 c2_23308 c2_23284
42.6 c2_23235 c2_23252 c2_23254 c2_23256 c2_23259 c1_11644 c2_39409 c2_39411 c2_50824 c2_48483
43.6 c2_57398 c2_57400
44.7 c2_32466 c2_32517 c2_32482
45.7 c2_32077
48.9 c1_2331
54.4 c2_8037
55.5 c1_2689 c1_2690 c2_46213 c1_1944
56.5 c1_1923
57.6 c2_5713
58.7 c1_14759 c1_1985
59.7 c2_57482 c2_57484 c2_5507 c2_5474 c2_5461 c2_5463 c2_5524
XII D84
0.0 c2_24563 c2_24586 c2_24558
2.4 c2_24454 c2_24466 c2_24432 c1_7744
3.1 c2_24595
3.9 c1_15641
6.3 c2_16238 c2_16200 c2_16204
7.1 c2_13645
10.2 c1_11730
11.8 c2_27928
13.4 c1_8631
14.1 c2_28006
15.7 c2_31301
16.5 c2_50983
17.2 c1_13762 c1_14538 c1_13754
18.0 c1_13767 c1_13809
18.8 c2_40699
21.2 c2_24655 c2_24645
21.9 c1_7770
22.7 c2_24646
23.5 c2_34807
24.3 c2_34780 c2_53931
25.8 c2_3185
26.6 c1_9646 c2_27379
27.4 c2_27398
29.7 c2_51300 c2_40748 c1_403 c1_14759 c2_14411 c2_40745 c2_16300 c1_15034 c2_45803 c1_14396
30.5 c2_48008
32.9 c2_17615
34.4 c2_18827 c2_52567 c2_42328 c2_53324 c1_6004
35.2 c2_23308 c2_23284 c2_23235 c2_23252 c1_11644 c2_50824 c2_39411 c2_48483 c2_57398 c2_32466 c1_7495 c2_51234
36.0 c2_32073
36.8 c1_2350
37.5 c2_6487
38.3 c2_7902
39.1 c2_7915
39.9 c2_8037 c1_2727
40.6 c1_2690
46.3 c1_15889
47.9 c2_46213
49.5 c2_5713
51.0 c1_1985 c2_5595
52.6 c2_5474 c2_5463 c2_5461 c2_5524 c2_5574
53.4 c2_5440
XII MSX902
0.0 c2_24586
0.4 c2_24563
0.8 c2_24454
1.2 c2_24432
1.6 c1_7744
2.0 c1_15641 c2_24595
7.5 c2_16238
8.6 c2_16200
8.9 c1_11730
9.7 c2_16204
11.6 c2_27959
12.3 c1_8631
12.9 c2_27931 c2_27960 c2_28006
14.0 c2_31301
15.8 c2_49199
16.2 c1_14538
17.2 c1_13762
18.6 c1_13767
19.6 c1_13809
20.0 c2_40699
21.7 c2_24655 c2_34780
22.5 c2_24645 c1_7770 c2_24646 c2_34807 c2_53931
23.4 c2_3185
25.9 c1_9646
26.2 c2_53918
26.9 c2_32120
27.6 c2_27379 c2_27398
29.5 c2_44932
29.7 c2_14411
30.0 c2_40748
30.1 c2_16300
30.3 c2_51099
30.8 c2_51300 c1_403 c1_15034
31.6 c2_45803
32.0 c1_14396
32.3 c2_18995
32.9 c2_48687
33.5 c2_48008
35.0 c1_6004
35.8 c2_17615
36.5 c2_18827
36.9 c2_17747
37.3 c2_52567
37.7 c1_14869
38.5 c2_18791
38.8 c2_42328
39.3 c2_18836
40.3 c2_53324
41.8 c2_23308
42.6 c2_23284
43.4 c2_23235
43.6 c2_23254
43.8 c2_23252
44.8 c2_39374
45.3 c1_11644
45.7 c2_50824
46.1 c2_39411
46.9 c2_48483 c2_57398
48.1 c2_32466 c2_32077 c2_32073
52.1 c2_6487
52.8 c1_2350
54.1 c2_7902 c2_7915 c1_2727
54.9 c2_8037
60.1 c2_50875
60.4 c1_15889
60.9 c1_13698
62.5 c1_1944
62.7 c2_5713
65.0 c2_5474 c2_5595
65.8 c2_5463
66.6 c1_1985
66.7 c2_5507
67.2 c2_5461
68.1 c2_57484 c2_5574 c2_5440
68.9 c2_5524

Fig. S2. Comparative map of DRH, D84 and MSX902 genetic maps. A total of 1612 SNPs that mapped to unique positions in the individual population maps, the common maker positions commonly mapped to all populations, and any combination of two populations.
