# Supplementary material for: Comparative Analysis of Regions with Distorted Segregation in Three Diploid Populations of Potato
Source: G3 (Bethesda). 2016 Jun 23;6(8):2617–28. doi: 10.1534/g3.116.030031 (PMC4978915; doi:10.1534/g3.116.030031)
Supplement: Supplemental Material [file supp_g3.116.030031_FigureS3.pdf]

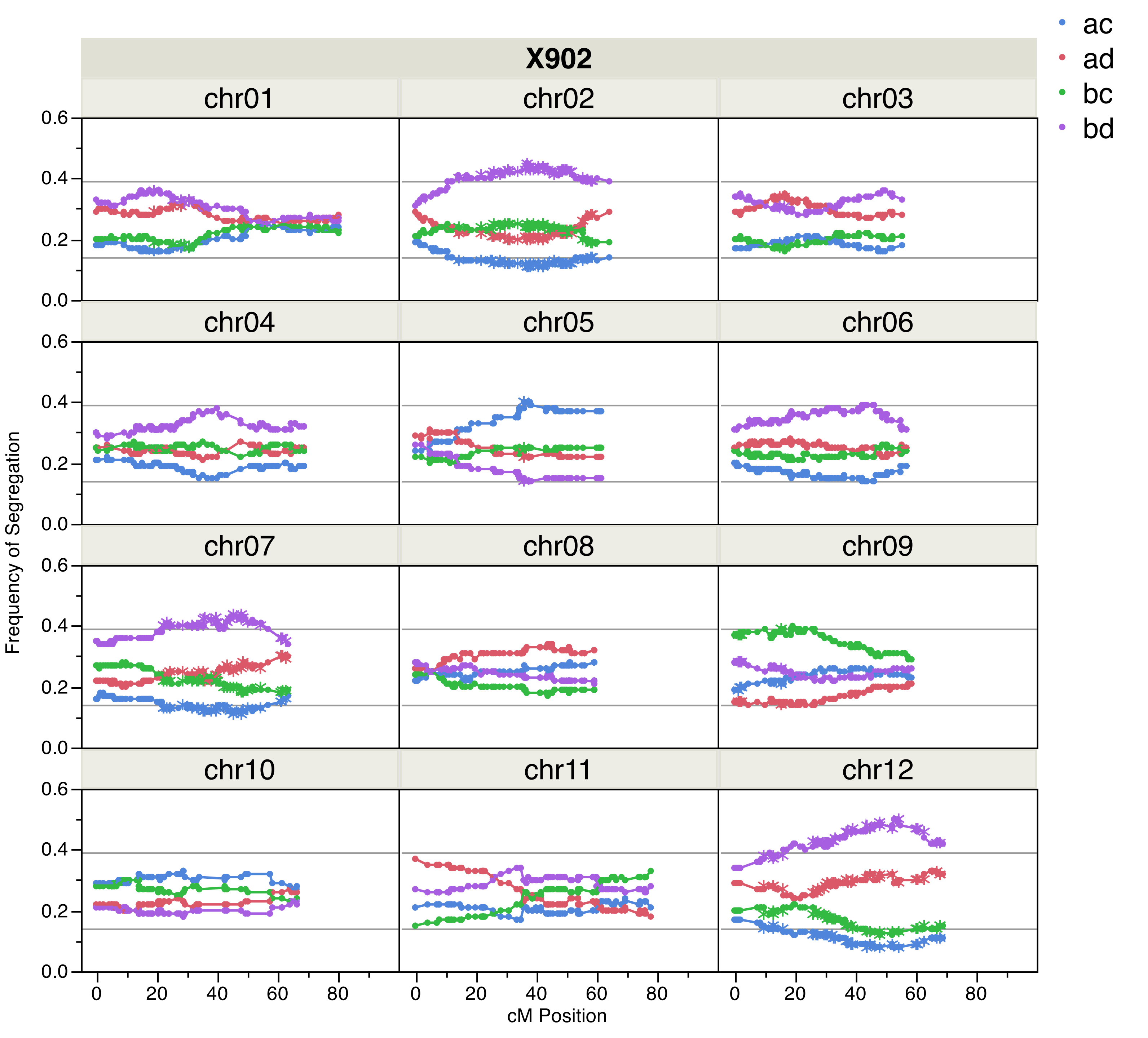
X902
chr01
chr02
chr03
chr04
chr05
chr06
chr07
chr08
chr09
chr10
chr11
chr12
ac
ad
bc
bd
Frequency of Segregation
cM Position
0.0
0.2
0.4
0.6
0
20
40
60
80

Fig. S3. Distribution of segregation ratios of parental haplotype combinations along the genetic linkage map (cM) for MSX902 population. The lines in each chromosome (chr) represent the confidence interval for a Chi-square test with α =0.1%. SNPs with distorted segregation represented by asterisks are located outside the confidence interval. Haplotype combinations 84SD22-1 and Ber83-1 (ac), 84SD22-1 and Ber83-2 (ad), 84SD22-2 and Ber83-1 (bc), 84SD22-2 and Ber83-2 (bd).
