# Supplementary material for: Comparative Analysis of Regions with Distorted Segregation in Three Diploid Populations of Potato
Source: G3 (Bethesda). 2016 Jun 23;6(8):2617–28. doi: 10.1534/g3.116.030031 (PMC4978915; doi:10.1534/g3.116.030031)
Supplement: Supplemental Material [file supp_g3.116.030031_FigureS7.pdf]

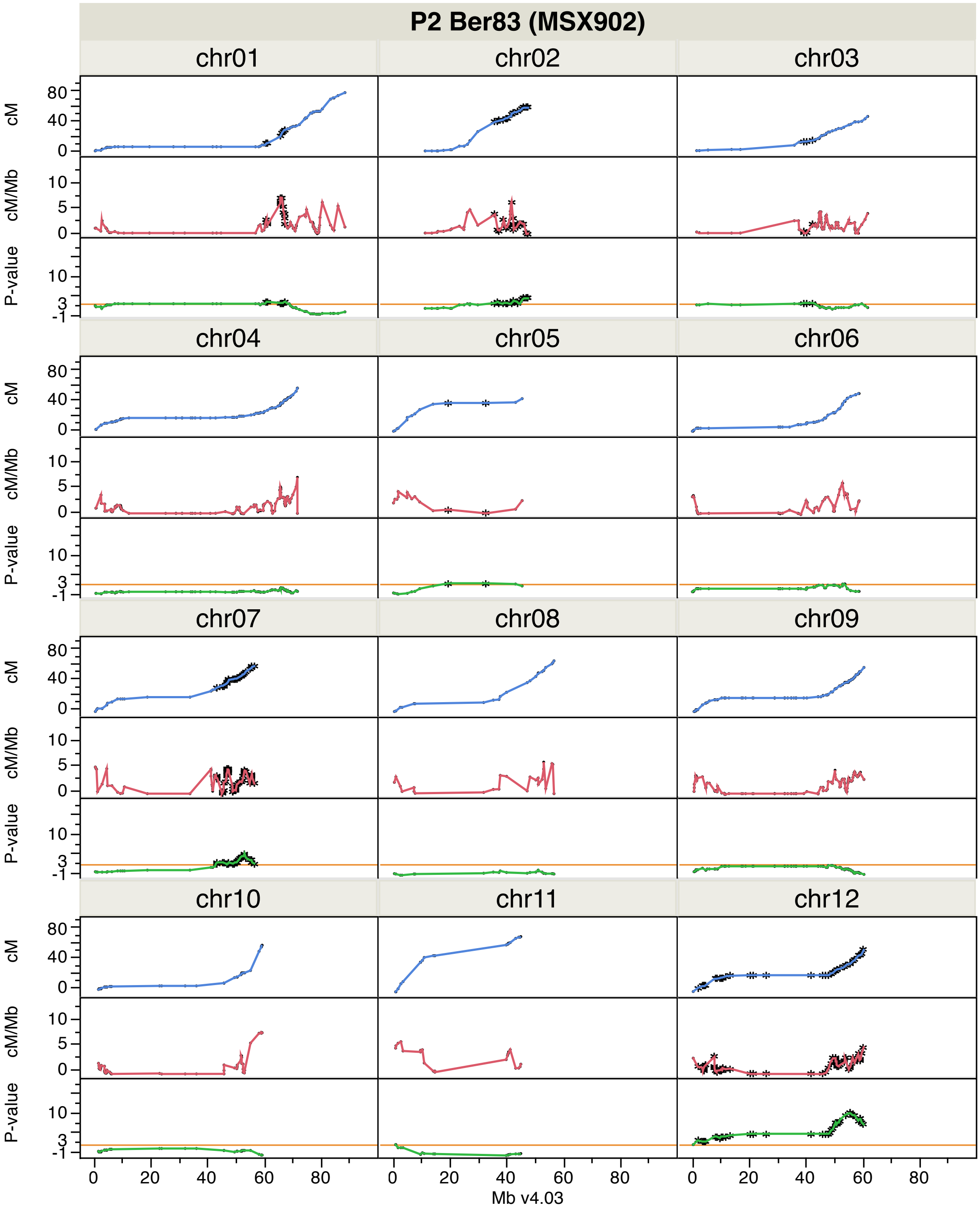

P2 Ber83 (MSX902)
chr01
chr02
chr03
chr04
chr05
chr06
chr07
chr08
chr09
chr10
chr11
chr12
cM
cM/Mb
P-value
Mb v4.03

Fig. S7. Distribution of recombination rates along chromosomes with distorted segregation regions for Ber83 female parent P2 of MSX902 population. For each chromosome (chr), in the upper panel is the Marey map, the middle panel is the recombination rate (cM/Mb), and the lower panel is the significance of distorted segregation reported as the minus logarithm of chi square test P-value (P-value), plotted against physical position in Mb based on potato genome assembly version 4.03 (Mb v4.03). The 0.1% threshold of significance used to define distorted segregation corresponds to orange line of 3. Black stars highlight loci with distorted segregation.
